# Supplementary material for: Rare and common vertebrates span a wide spectrum of population trends
Source: Nat Commun. 2020 Sep 2;11:4394. doi: 10.1038/s41467-020-17779-0 (PMC7468135; doi:10.1038/s41467-020-17779-0)
Supplement: Supplementary file 1 — Supplementary Information [file 41467_2020_17779_MOESM1_ESM.pdf]

Daskalova *et al.*

"Rare and common vertebrates span a wide spectrum of population trends"

Supplementary Information

* Corresponding author:
Gergana N. Daskalova
gndaskalova@gmail.com
Crew Building, King's Buildings
Edinburgh EH9 1UU
Scotland

This PDF file includes:

- Supplementary Tables 1 to 8
- Supplementary Figures 1 to 18

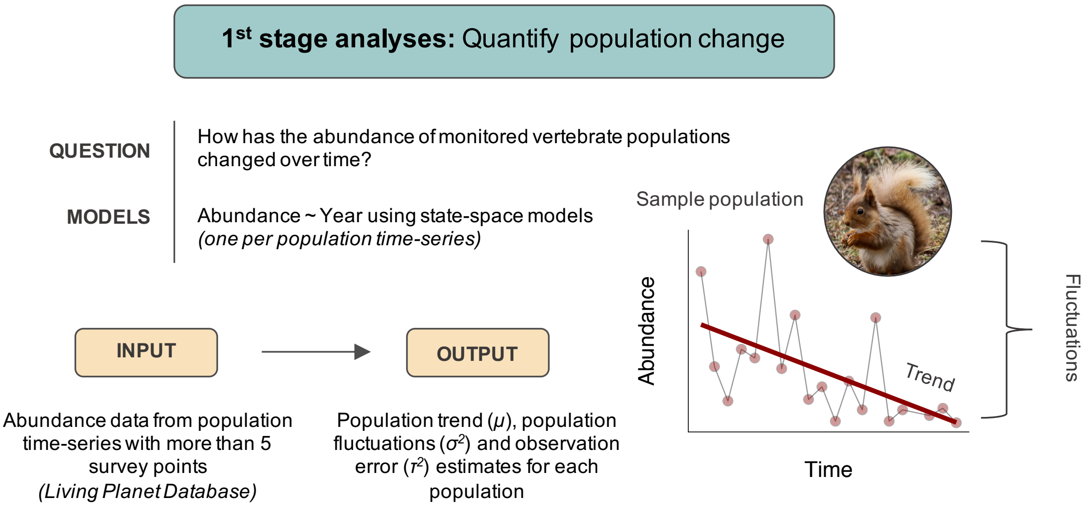

Supplementary Figure 1. Conceptual diagram of the first stage of our analyses where we calculated population trends and fluctuations. We analysed vertebrate population time-series from the Living Planet Database (133,092 records) covering the period between 1970 and 2014. These time-series represent repeated monitoring surveys of the number of individuals in a given area (species' abundance over time), to which we refer as "populations". Diagram shows one sample population of Red squirrel (*Sciurus vulgaris*). We quantified two aspects of population change – overall change in abundance over time (population trends) and abundance variability over time (population fluctuations). We used state-space models that account for observation error and random fluctuations[1]. The input abundance data for the state-space models were scaled to a common magnitude between zero and one to analyse within-population relationships to prevent conflating within-population relationships and between-population relationships[2]. See methods for additional details. Squirrel photo by G. Daskalova.

2nd stage analyses: Test heterogeneity in population trends and fluctuations

Prior structure 1:
Hierarchical models in a Bayesian framework with weakly informative (flat) priors

$Pr(\mu) \sim N(0, 10^8)$
$Pr(\sigma^2) \sim Inverse\ Wishart\ (V = 0,\ nu = 0)$

Prior structure 2:
Hierarchical models in a Bayesian framework with weakly informative (parameter expanded) priors and a variance-covariance structure that allows the slopes of population trends and fluctuations to covary for each random effect.

$Pr(\mu) \sim N(0, 10^8)$
$Pr(\sigma^2) \sim Inverse\ Wishart\ (V = 1,\ nu = 1)$

INPUT

Population trend (μ), population fluctuations (σ^2) and observation error (τ^2) estimates for each population

GEOGRAPHIC PATTERNS

QUESTIONS How do vertebrate population trends and fluctuations vary across latitudes, realms and biomes?

MODELS

μ ~ latitude, random = species
μ ~ 1 + realm, random = species
μ ~ 1 + biome, random = species
σ² ~ latitude, random = species
σ² ~ 1 + realm, random = species
σ² ~ 1 + biome, random = species

+

Bird, amphibian and reptile phylogenies

TAXONOMIC PATTERNS

QUESTIONS How do vertebrate population trends and fluctuations vary across taxa and phylogenies?

MODELS

μ ~ 1 + taxa, random = species
σ² ~ 1 + taxa, random = species
μ ~ 1, random = species + phylogeny
σ² ~ 1, random = species + phylogeny

Prior structure 2 (applies to the phylogeny models)

Prior structure 1 (applies to the geographic and taxa models above)

+

Bird and mammal geographic range, species' mean population size, species' habitat specificity

RARITY PATTERNS

QUESTIONS How do vertebrate population trends and fluctuations vary across rarity metrics?

MODELS

μ ~ log(geographic range), random = species
μ ~ log(mean population size), random = species
μ ~ habitat specificity, random = species
σ² ~ log(geographic range), random = species
σ² ~ log(mean population size), random = species
σ² ~ habitat specificity, random = species

+

Species' IUCN conservation status and IUCN threats categories

IUCN CONSERVATION STATUS AND TREATS

QUESTIONS How do vertebrate population trends and fluctuations vary across species' IUCN Red List Categories and type and number of threats?

μ ~ 1 + IUCN Red List Category, random = species
μ ~ 1 + threat type, random = species
μ ~ number of threats, random = species
σ² ~ 1 + IUCN Red List Category, random = species
σ² ~ 1 + threat type, random = species
σ² ~ number of threats, random = species

Prior structure 1 (applies to the rarity and IUCN models)

OUTPUT

Effect sizes for each tested metric

Supplementary Figure 2. Conceptual diagram of the second stage of our analyses where we quantified the geographic, taxonomic, rarity and threat patterns within vertebrate population trends and fluctuations. We modelled the trend and fluctuation estimates from the first stage (Supplementary Figure 1) across latitude, realm, biome, taxa, rarity metrics, phylogenetic relatedness, species' conservation status and threat type using a Bayesian modelling framework[3]. Each model included a species random intercept effect to account for the possible correlation between the trends of populations from the same species. The prior structure (weakly informative priors) was identical across all models except the phylogeny models from the taxonomic patterns section, where the prior structure allowed for an additional phylogeny random effect. See methods for additional details.

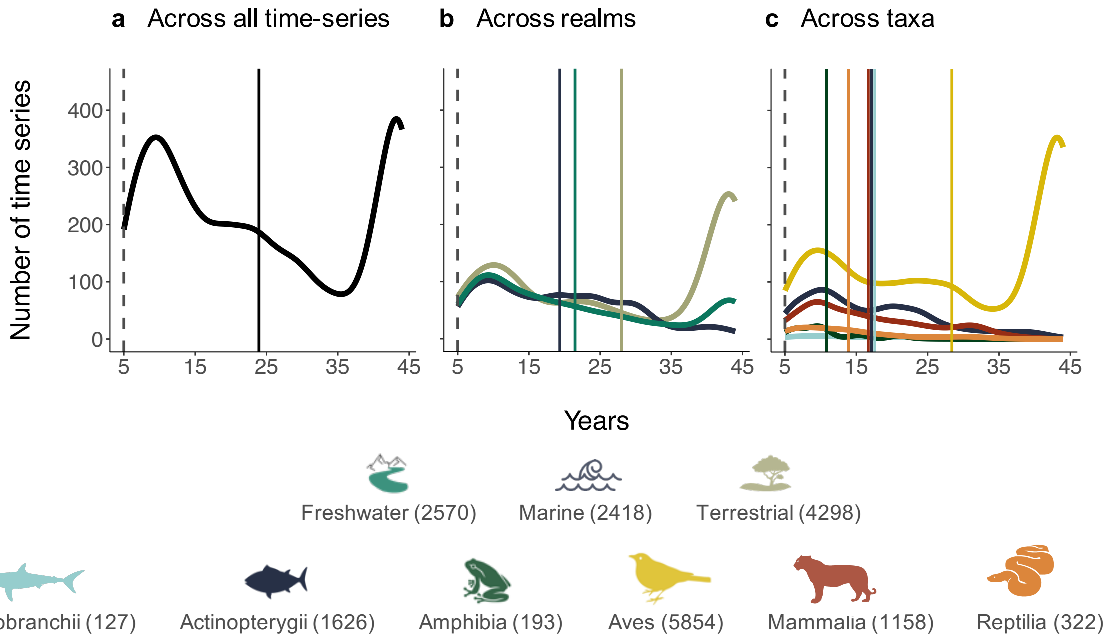

Supplementary Figure 3. The duration of monitoring varies by realm and taxa. Distribution of monitoring duration across (a) all time-series, (b) realms and (c) taxa. In our study, we included time-series with more than five survey points in time, with the dashed line representing five years and solid lines showing the mean duration for each category. Numbers in legend correspond to sample size in each category. Icon credits: tree by FayraLovers, wave by Setyo Ari Wibowo, mountain and stream by Nikita Kozin, bird by Hernan D. Schlosman, snake and frog by parkjisun, fish by Julia Söderberg.

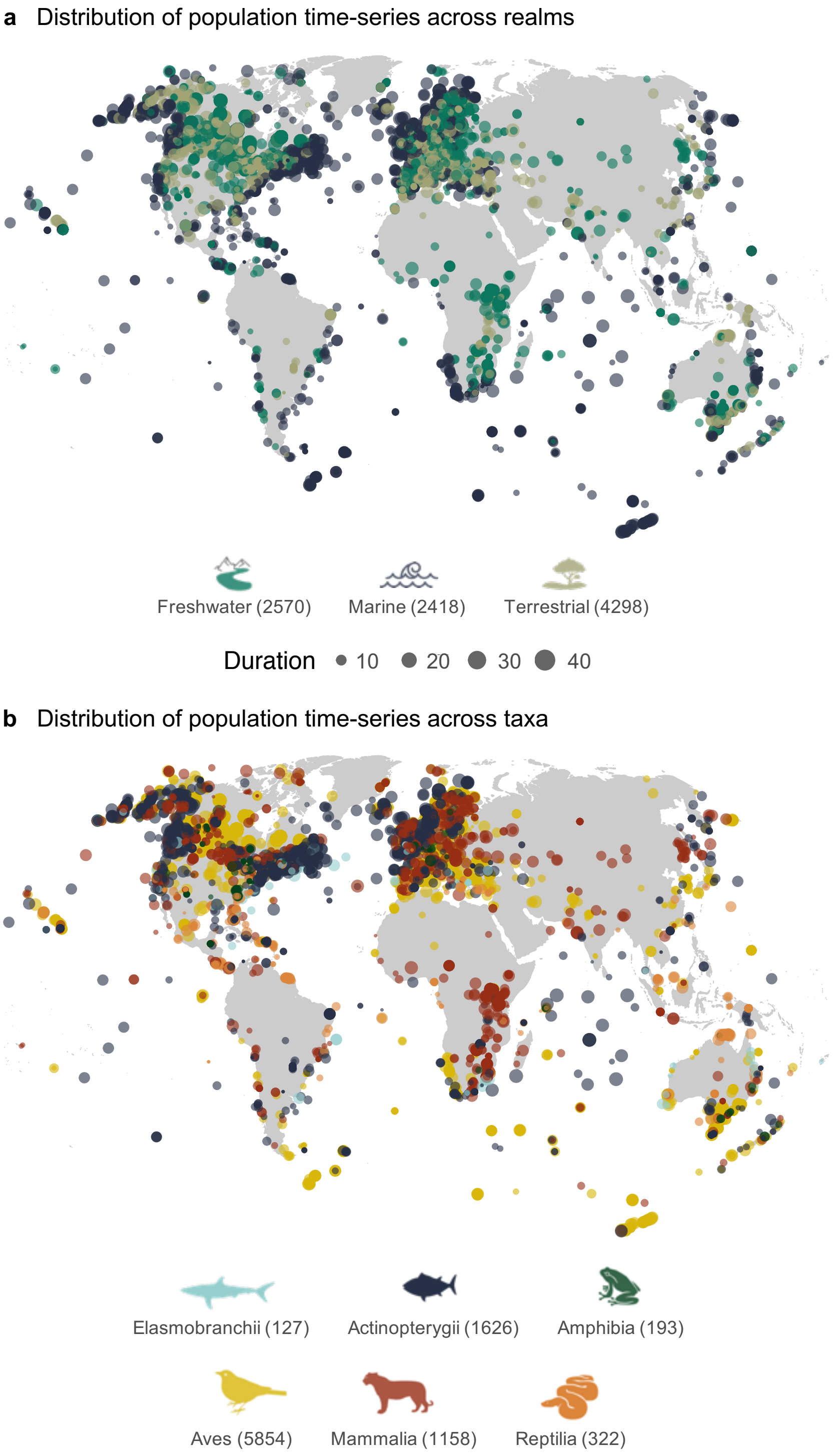

Supplementary Figure 4. The Living Planet Data represents a broad range of geographic locations, ecological settings and taxonomic groups. Our analysis of the patterns in vertebrate population trends and fluctuations includes time-series across realms (a) and different taxa (b), with a global geographic distribution of records. Numbers in legend correspond to sample size in each category. Icon credits: tree by FayraLovers, wave by Setyo

Ari Wibowo, mountain and stream by Nikita Kozin, bird by Hernan D. Schlosman, snake and frog by parkjisun, fish by Julia Söderberg.

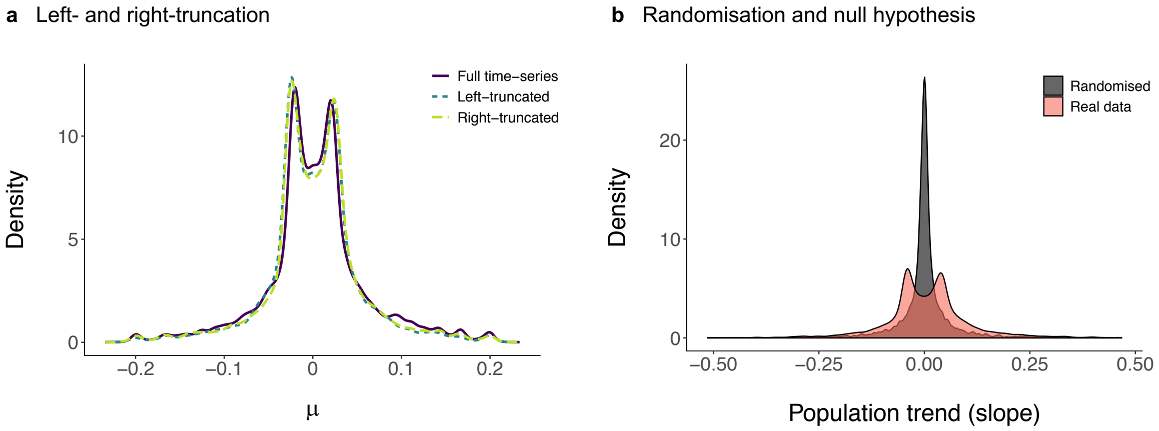

Supplementary Figure 5. The distribution of population trend values across time-series was not sensitive to the omission of the first five (left-truncation) or the last five years (right-truncation) of population records and it differed from a null distribution derived from randomised data. Following Fournier *et al.* 2019[4], we tested the time-series that we analysed for site-selection bias. Removing the first five survey points reduces the bias stemming from starting population surveys at points when individual density is high, whereas removing the last five years reduces the bias of starting surveys when species are very rare. There were slightly fewer trends centred on zero (no net change in abundance over time) when we left- and right-truncated the data, suggesting that longer time-series are more likely to show no net changes in abundance (see Supplementary Figure 6 for a visualization of population trends versus monitoring duration. We also compared the distribution of estimated population trends against a null hypothesis (b). To derive a null distribution, we used a randomisation approach. Within each time series, we randomised the abundance data, keeping the overall range of the original data. The two peaks of μ are apparent in the overall distribution of time series data. These peaks are created by many weakly positive and negative population trends from longer time series that often are bird species from terrestrial systems. We hypothesise that there might a publication bias against no net change studies, or a bias against including such studies in global databases.

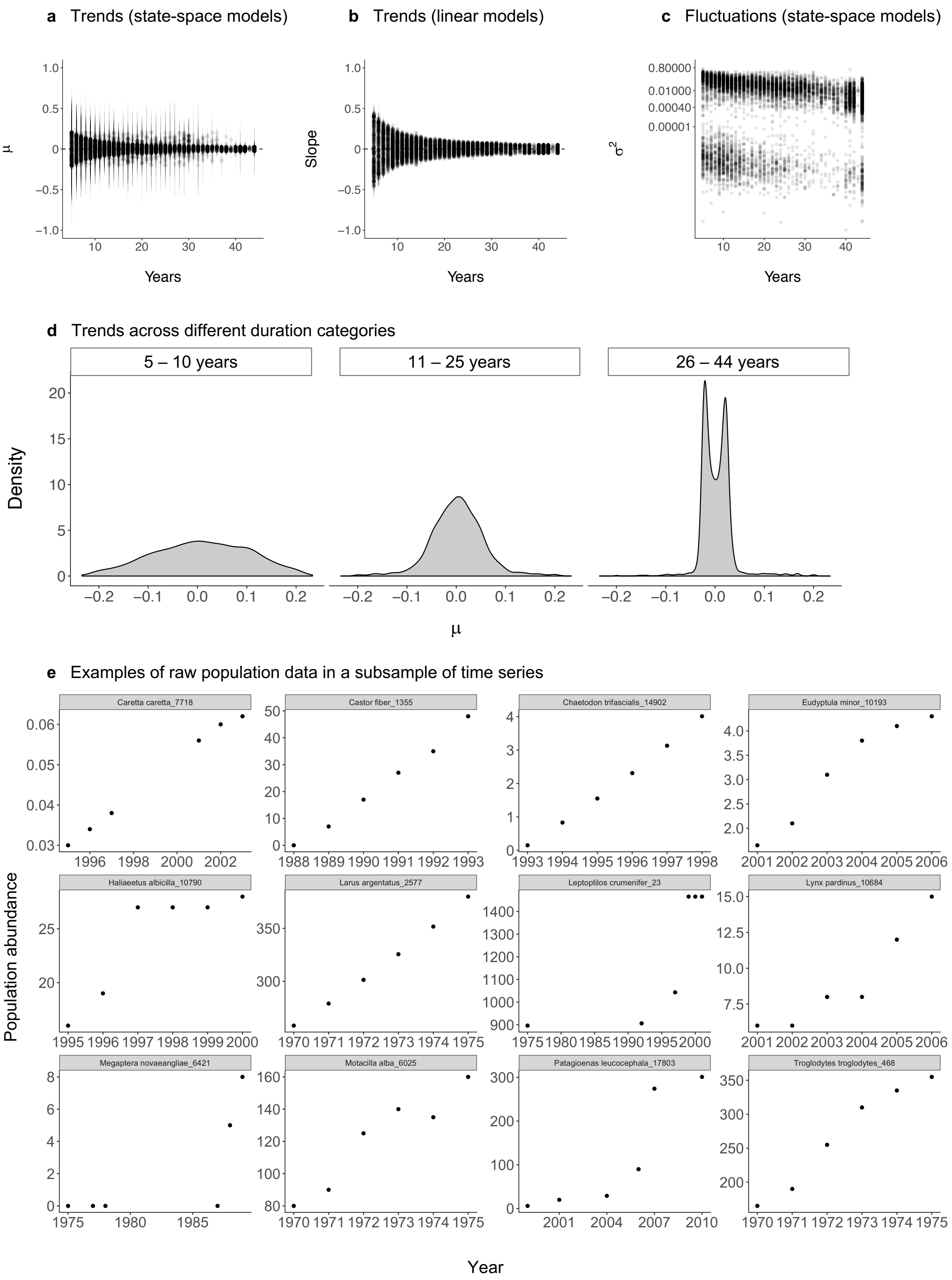

Supplementary Figure 6. Both positive and negative vertebrate population trends were smaller in magnitude for longer time-series of data. Monitoring duration results are for 9286 populations from 2084 species. Population trends (*μ*) were estimated for all populations

monitored for more than five time points using state-space models (a, d) and linear models (b). Population fluctuations (c) are plotted on a log10 y axis and represent the estimates for process noise (σ^2, the process noise is the total variance around the population trend minus the variance attributed to observation error) derived from state-space models. Error bars on (a) and (b) show 95% confidence intervals and their centres show population trends from state-space models (a) and linear models (b). The sample sizes for the duration categories were as follows 5 - 10 years: 2084 time series; 10 - 25 years: 3358 time series; 25 - 44 years; 3844 time series. Plot (e) shows the raw population trend data behind 12 time series which had the same population trend values (μ = 0.20). These time series are part of a "band" of time series which had very similar population trend estimates. Eighty, or approximately 1% of the time series we analysed form linear relationships over time with errors around the slopes of <0.001, such that we suspect these data might be modelled rather than measured population data. The presence of modelled data within the dataset may help partially explain the low variance bands of σ^2 values (c) and the pattern of two peaks in weak population increases and decreases for longer time series (d). Please see Methods sections "Time series with low variation" and "Clustering in the values of population trends and fluctuations" for further details.

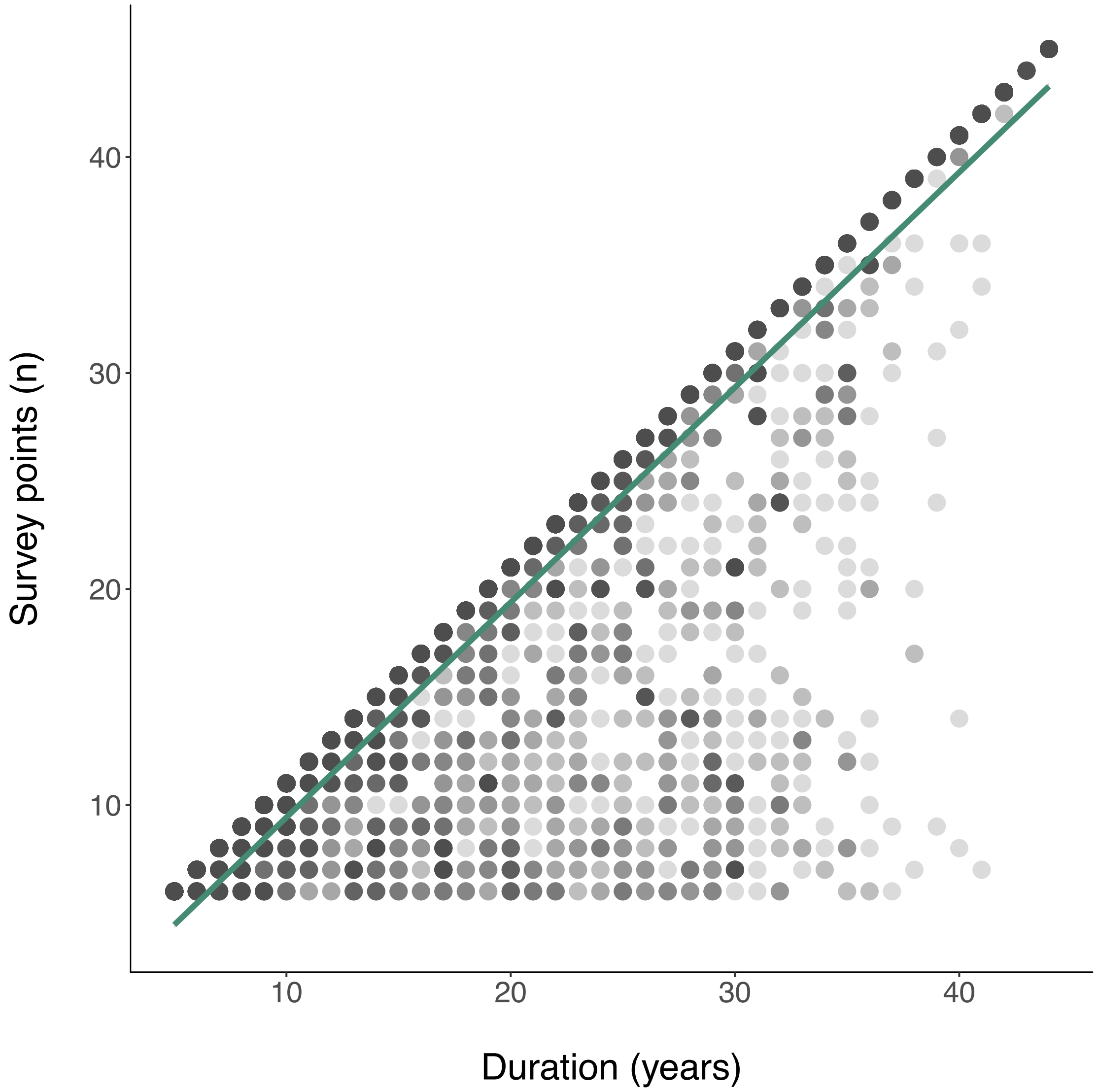

Supplementary Figure 7. Number of survey points within time-series positively correlates with time-series duration. We included time-series with more than five survey points in time in our analyses, but populations were not always monitored in each intervening year. Green line shows a linear model fit of survey points versus duration. There was a minimum of six time points for each time-series. Among the time-series we analysed, 18% had a duration of less than 10 years, 30% had a duration between 10 and 20 years, 18% had a duration between 21 and 30 years, and 33% had a duration between 31 and 44 years. See Supplementary Figure 3 for the density distribution of monitoring duration for the studies we included in our analyses.

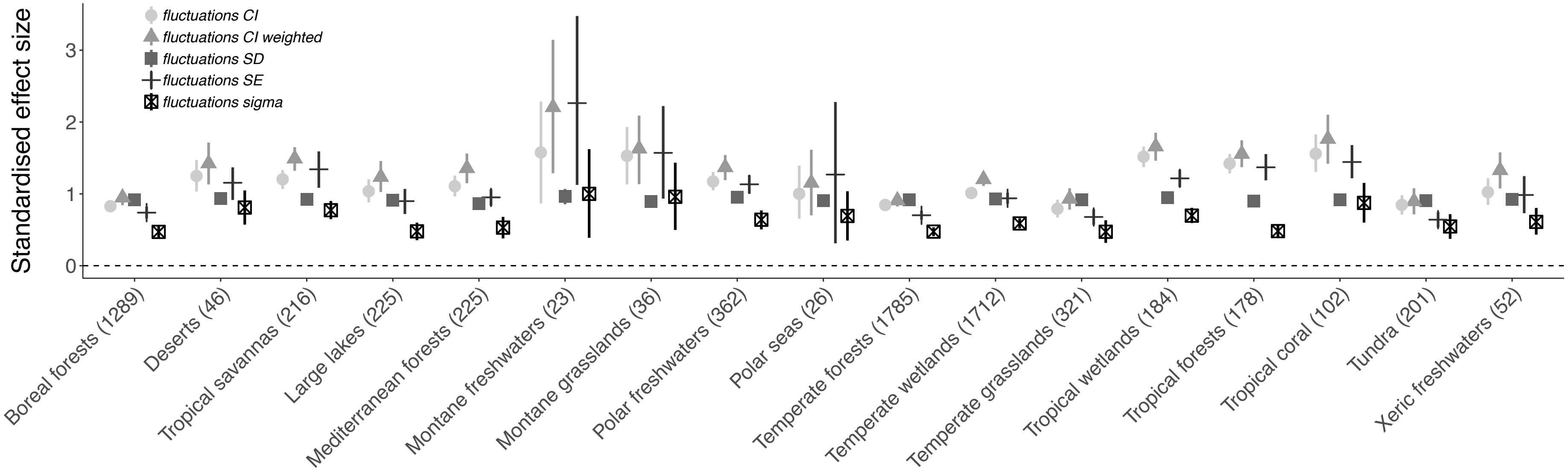

Supplementary Figure 8. Population fluctuations did not show distinct biome-specific patterns, with the exception of montane and tropical biomes where fluctuations were more pronounced compared to the rest of the biomes we studied. The five estimates (centre points of error bars) for each category refer to different analytical approaches, where the response variables in the models were: 1) the standard error around the slope estimates of the linear model fits of abundance versus year (circles), 2) half of the 95% confidence interval around the μ value of population change (triangles), 3) half of the 95% confidence interval around μ weighted by τ^2, (full squares), 4) the process noise (σ^2) from the state-space models, and 5) the standard deviation of the raw data for each population time-series (empty squares). The process noise is the total variance around the population trend minus the variance attributed to observation error. The effect sizes were standardized by dividing the effect size by the standard deviation of the corresponding input data. Error bars show 95% credible intervals.

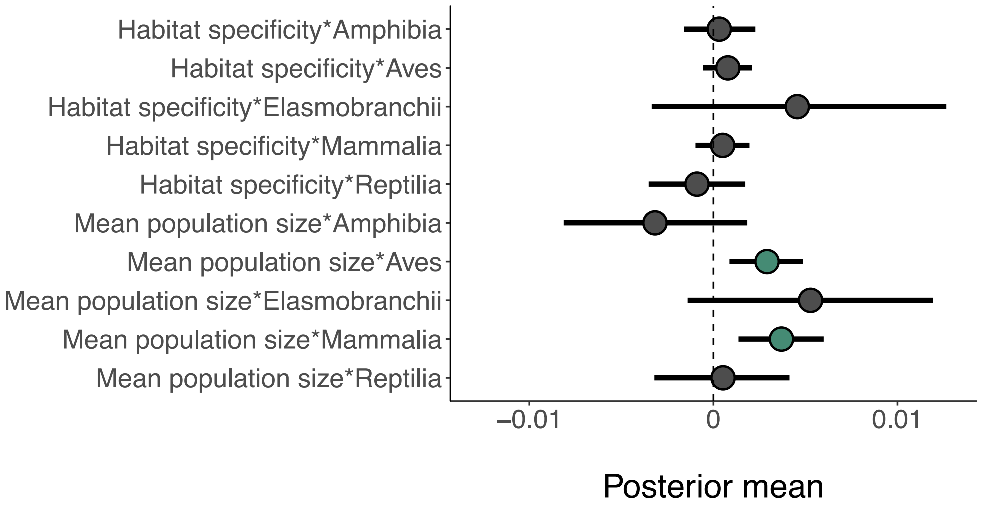

Supplementary Figure 9. Birds and mammals with larger mean population sizes are more likely to experience population increases. We tested for interaction effects of rarity and taxa on population trends and, with the exception of mean population size for mammals and birds, rarity traits were not significant predictors of population change. Teal colour indicates posterior means where the 95% credible intervals did not overlap zero, grey colour indicates the opposite. Error bars show 95% credible intervals and their centres show effect sizes. The sample size was 7901 population time series for the habitat specificity model and 4310 population time series for the mean population size model.

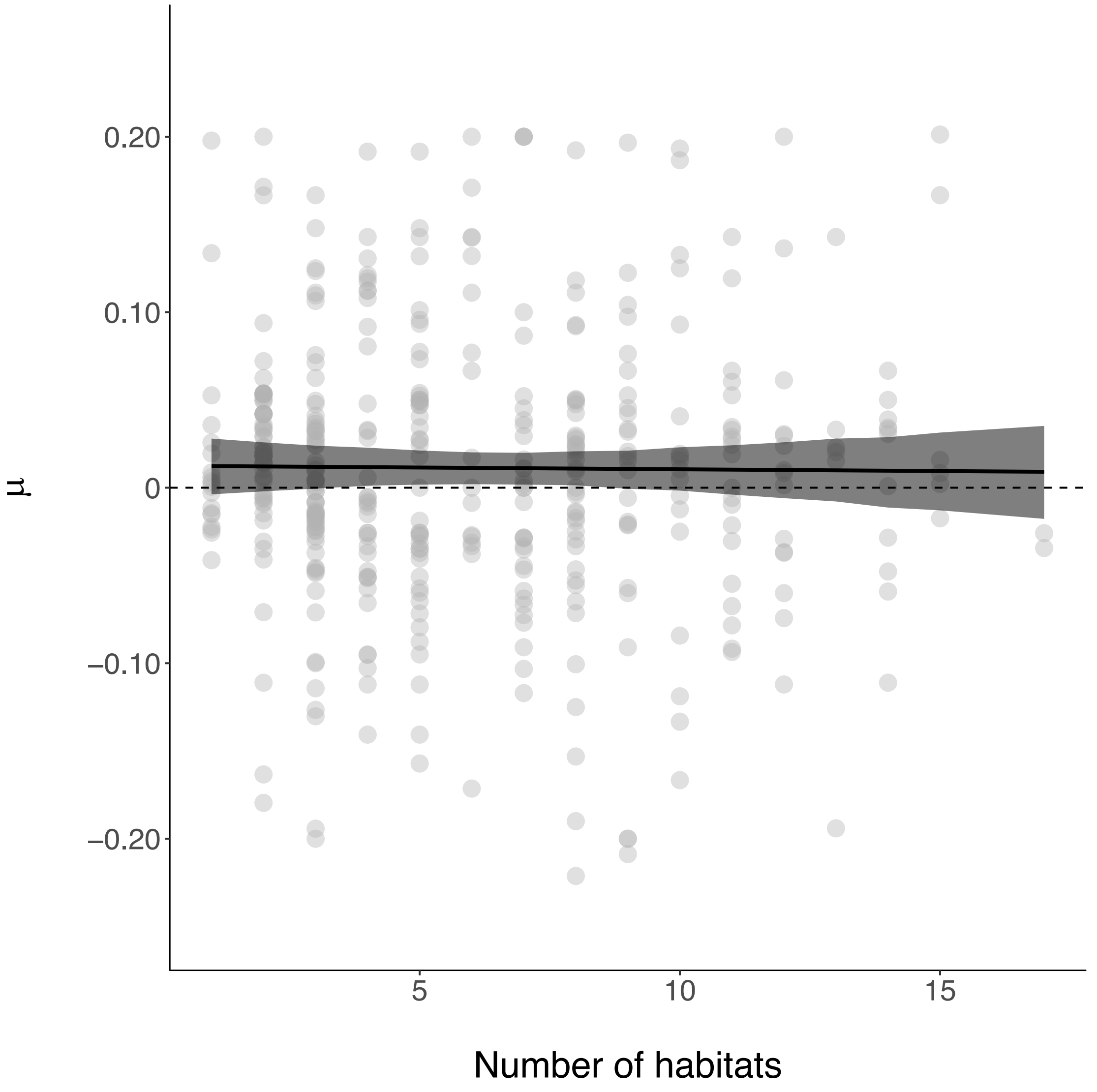

Supplementary Figure 10. Variation in vertebrate population trends was not explained by habitat specificity. Habitat specificity was calculated as the number of different habitats occupied by each species which we derived by surveying the 'Habitat and Ecology' profile for each species on the IUCN Red List website. The μ values of population change are derived from state-space model fits of changes in abundance over the monitoring duration for each population. Line shows model fit and shaded area shows 95% credible intervals. See Supplementary Table 2 for full model outputs. This figure is based on populations monitored in the UK, see Figure 3c for the effects of habitat specificity on population trends across bird and mammal species globally.

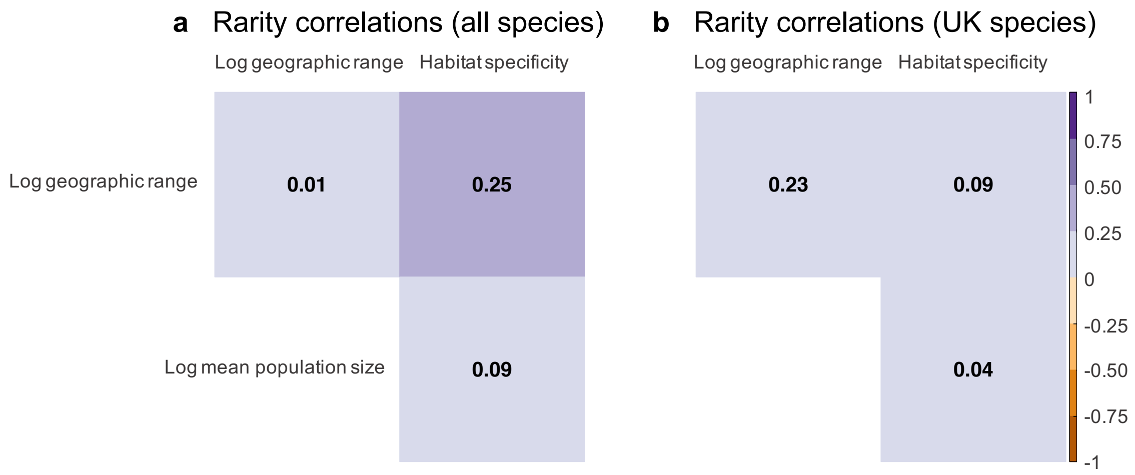

Supplementary Figure 11. The three rarity metrics used in this study were weakly correlated at both UK and global scales. See Supplementary Table 1 for sample sizes on each geographic scale.

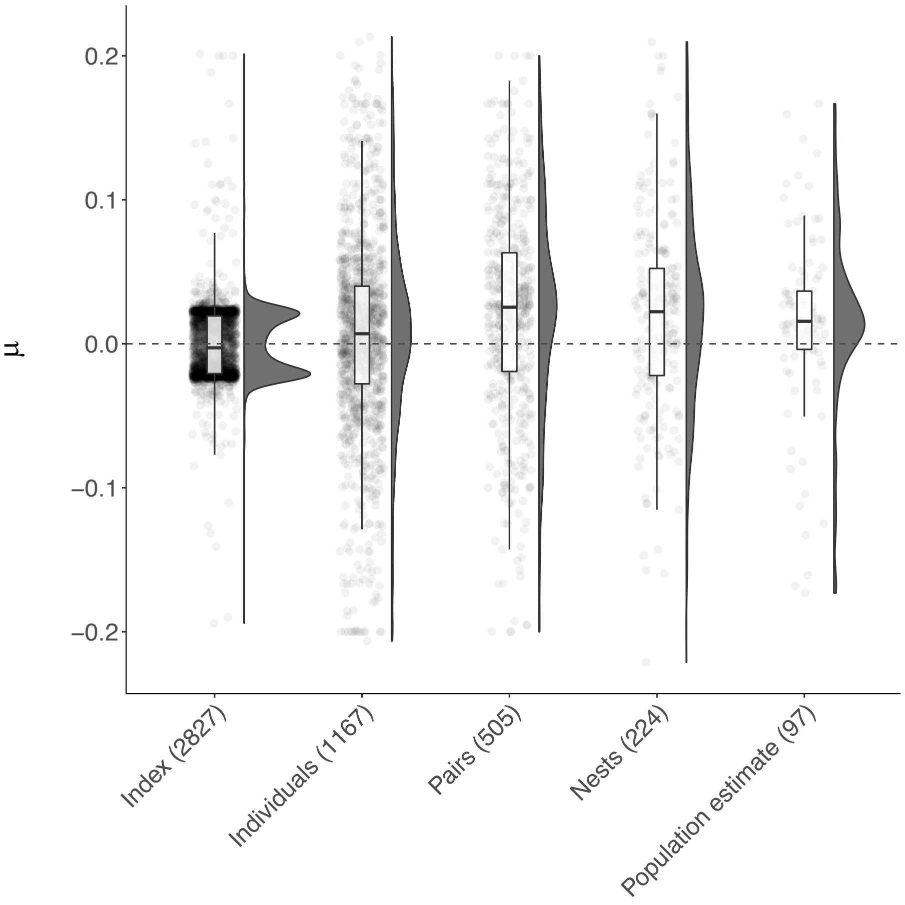

Supplementary Figure 12. There was no systematic difference in the distribution of population trends across commonly used abundance metrics in the Living Planet Database. Population trends represent μ values from state-space Numbers on the x axis correspond to sample size in each category. Density plots show the density distribution of population trends across sampling units, points show the raw values and boxplots show the mean, first and third quartiles and boxplot whiskers that cover 1.5 times the interquartile range. Population data where the units are an index were more likely to have weakly increasing or decreasing trends with many populations with μ values around 0.025 and -0.025. These μ estimates are reasonable trend estimates for these time series; however, there seem to be population within the Living Planet Database that are modelled, particularly for studies with longer duration which could partially explain these peaks in the population trend estimates near zero (see Figures 1, 2 and Supplementary Figures 5 and 6).

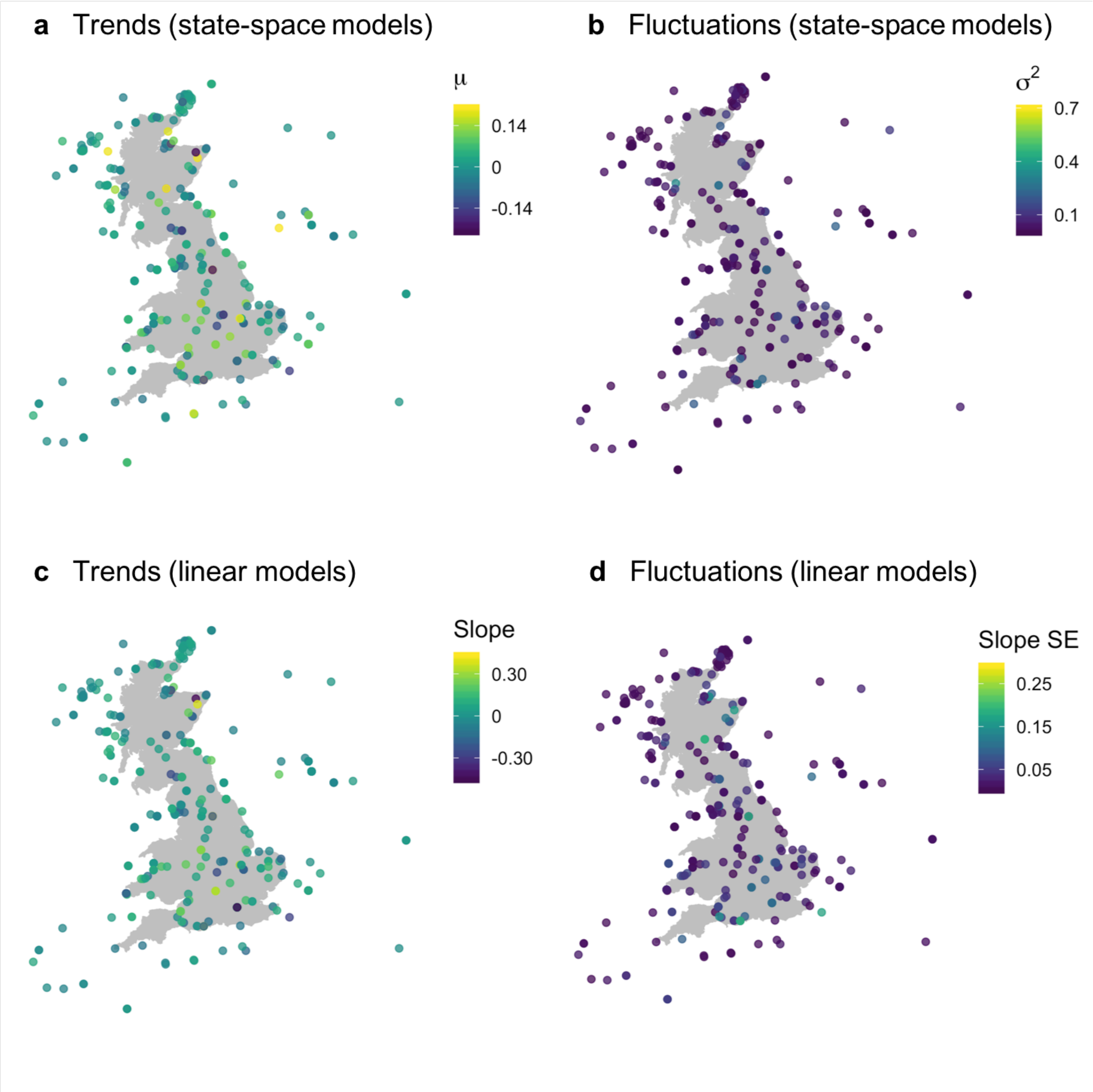

Supplementary Figure 13. Population trends and fluctuations did not show geographic patterning within the UK. Results include 508 populations from 237 species in the UK. See methods for additional details on the different ways we quantified trends and fluctuations.

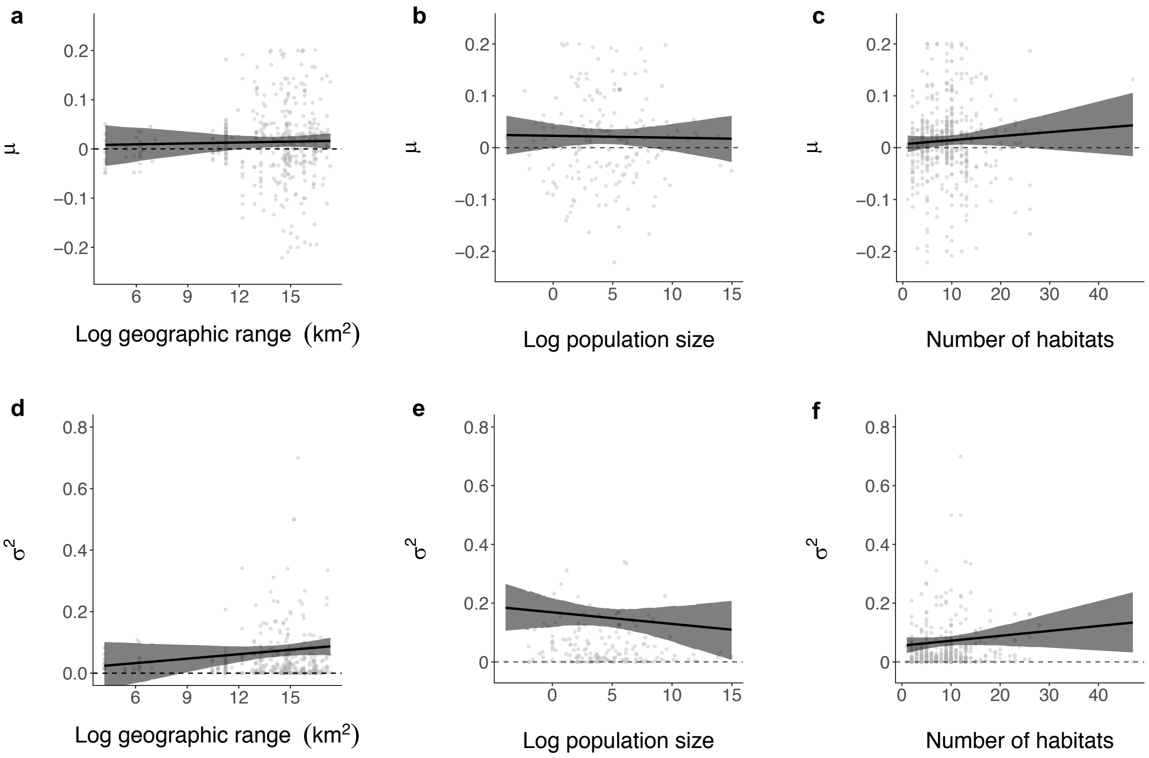

Supplementary Figure 14. The effects of rarity on population trends and fluctuations were consistent at both UK (pictured here) and global scales (Figure 3). Rare species were not more likely to decline than common species. Populations from species with smaller mean population sizes and populations from habitat generalist species were more likely to fluctuate. The μ values of population trend (plots **a-c**) and the σ^2 values of population fluctuation (d-f) were derived from state-space model fits of changes in abundance over the monitoring duration for each population. The population fluctuations represent the process noise from the state-space models which is the total variance around the population trend minus the variance attributed to observation error. Lines on plots a-f show model fits and 95% credible intervals. See Supplementary Table 1 for sample sizes for each analysis and Supplementary Table 3 for model outputs.

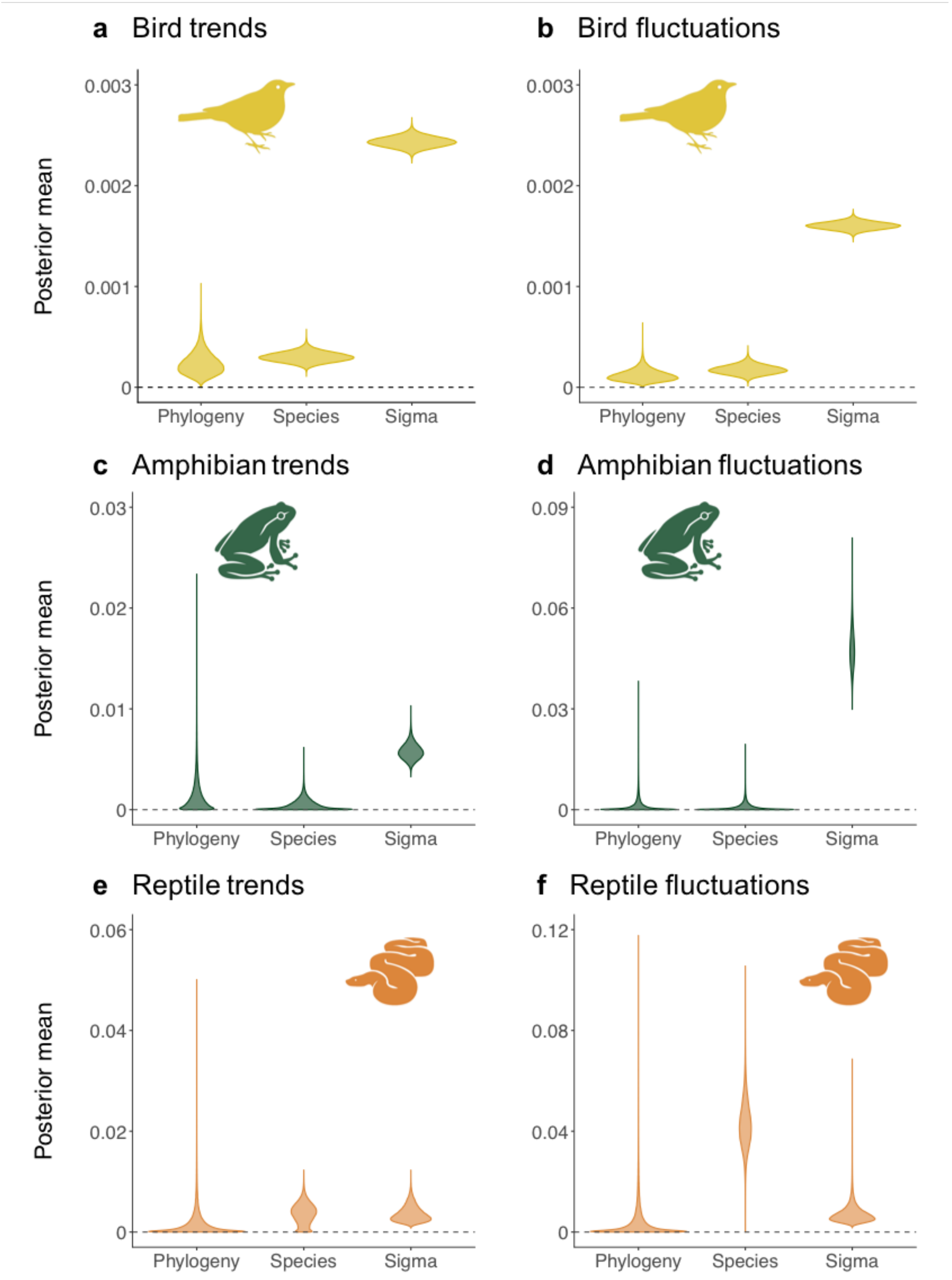

Supplementary Figure 15. There were no phylogenetic patterns in the population trends and fluctuations of birds, amphibians and reptiles. Phylogeny refers to variance explained by phylogenetic relationships, species refers to variance explained by within-species differences (some species were represented by more than one population, thus introducing species-level variance), and residual variance refers to the variance not explained by

phylogeny and species effects. The figure shows violin plots of the distributions of posterior means for phylogenetic, species and residual variance across taxa (the wider the violin, the more records there are with that value). The distributions are based on ten models for each taxon for each type of population change using 10 random trees to account for phylogenetic uncertainty. Phylogeny effects were calculated based on a branch length covariance matrix. See Supplementary Table 4 for full model outputs. Icon credits: bird by Hernan D. Schlosman, snake and frog by parkjisun, fish by Julia Söderberg.

a Population trends from state-space models

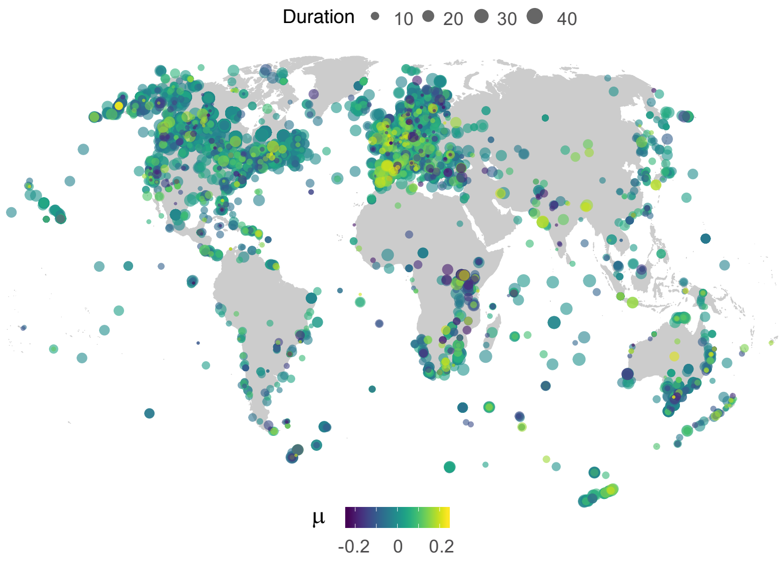

b Population trends from linear models

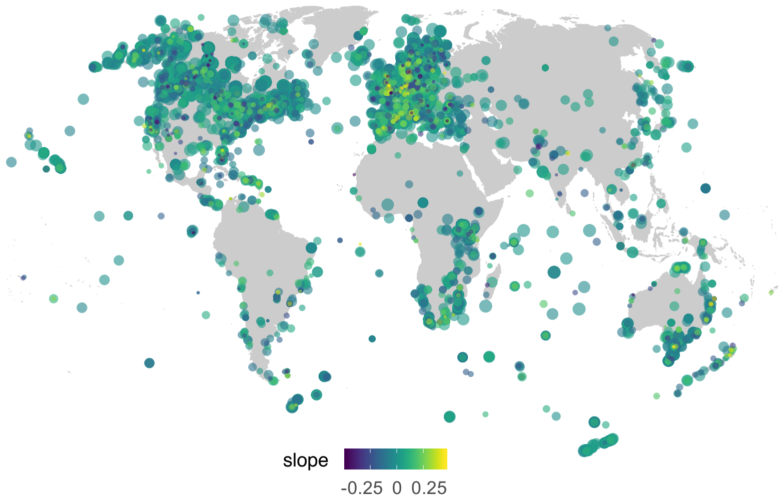

c Population fluctuations from state-space models

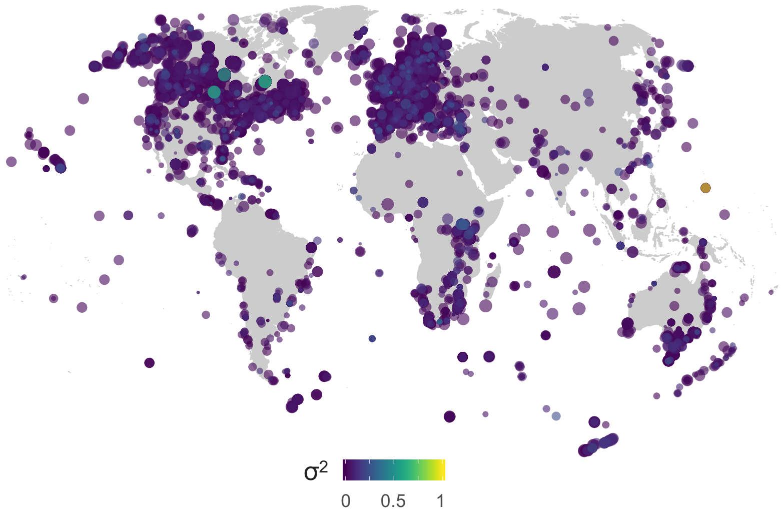

Supplementary Figure 16. Population change is ubiquitous across the planet, with no distinct hotspots of declines, increases or fluctuations. Maps show geographic distribution of time-series colour-coded by the magnitude of change experienced. The population fluctuations represent the process noise from the state-space models which is the total variance around the population trend minus the variance attributed to observation error. See methods for additional details on calculating trends and fluctuations.

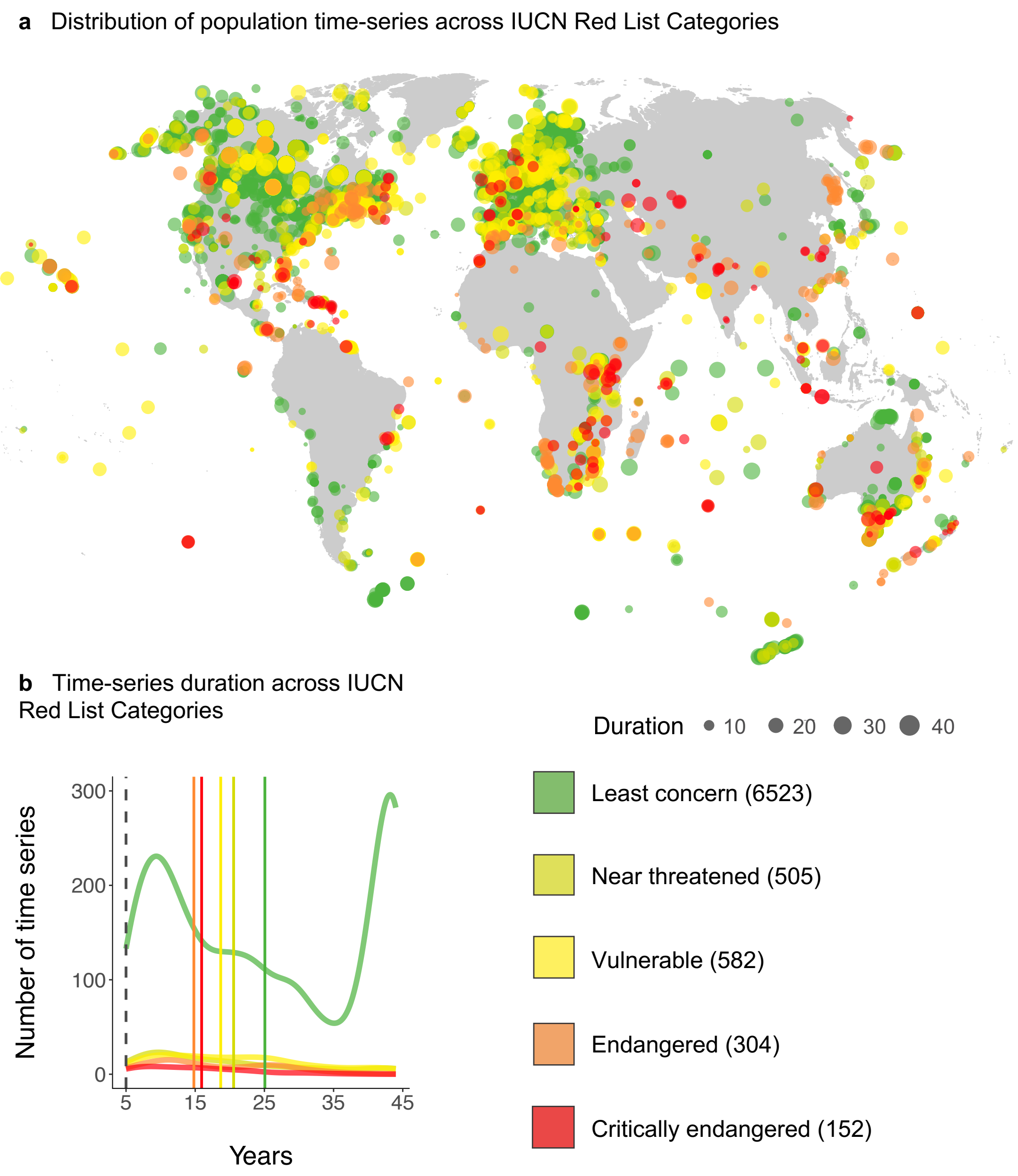

Supplementary Figure 17. Species across the whole spectrum of the IUCN Red List Categories are distributed around the world, with a concentration of least concern species in Northern America and Europe. In our study, we included time-series with more than five survey points in time (b), with the dashed line five years and solid lines showing the mean duration for each category. Populations from least concern species were monitored for longer durations. Numbers next to each category show sample size.

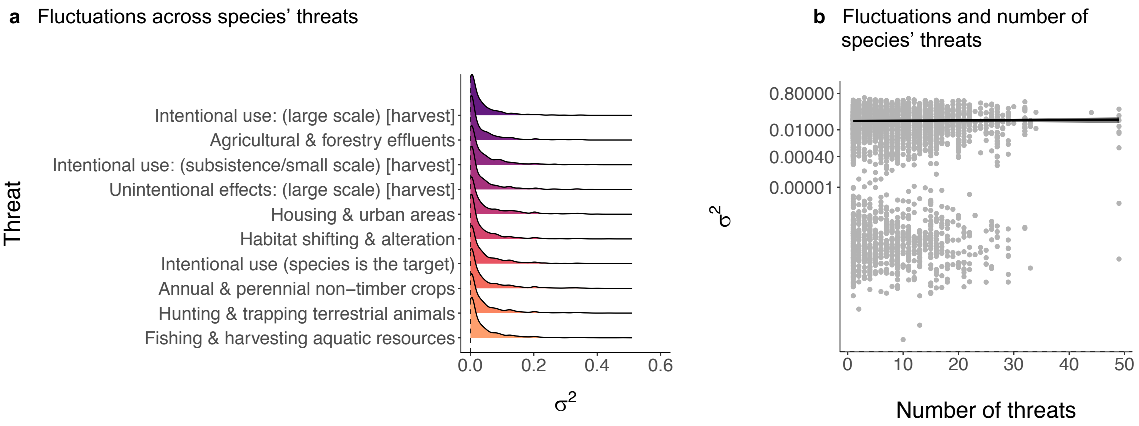

Supplementary Figure 18. Population fluctuations did not differ based on the type and number of threats based on species' IUCN Red List profiles. Fluctuations were estimated using the process noise (σ^2) values from state-space model fits of changes in abundance over the monitoring duration for each population. Densities in (a) show distributions of fluctuation estimates across different threats. Line in (b) shows model fit and shaded area shows 95% credible intervals, where "number of threats" refers to the number of different threats that each species, whose populations are locally monitored, could be exposed to throughout their distribution range, based on species' IUCN Red List profiles. See Methods for how the types of threats were derived and Supplementary Table 2 for model outputs. The sample sizes in (a) (number of population time series) were as follows: Intentional use: (large scale) [harvest] – 530, Agriculture and forestry effluents – 790, Intentional use: (subsistence/small scale) [harvest] – 567, Unintentional effects: (large scale) [harvest] – 807, Housing & urban areas – 730, Habitat shifting and alteration – 1331, Intentional use (species is the target) – 1208, Annual & perennial non-timber crops – 917, Hunting & trapping terrestrial animals – 1777, fishing & harvesting aquatic resources – 1514. The sample size in (b) was 3501 population time series.

Supplementary Table 1. Number of species and populations included in analyses.

Scale	Analysis	Number of species	Number of populations	Number of species with >3 populations
**Global**	System	2074	9286	834
	Biome	2074	9286	834
	Taxa			
	- *Actinopterygii*	544	1626	151
	- *Amphibia*	78	193	21
	- *Aves*	968	5852	538
	- *Mammalia*	306	1158	94
	- *Reptilia*	110	322	16
	Red List status	1702	8064	708
	Latitude	2074	9286	834
	Duration	2074	9286	834
**UK**	Geographic range	167	381	32
	Population size	112	253	19
	Habitat specificity	144	289	29
	Red List status	153	364	31

Supplementary Table 2. Model outputs from global analyses. Sigma is the overall model residual variance. Net population change is estimated using μ values derived from state-space models of population abundance versus time and slopes of linear models of population abundance versus time. The weighted μ models included μ as a response variable, weighted by τ, the observation error estimate derived from the state-space models. The fluctuation models included the process noise (σ^2) values from state-space models, half of the 95% confidence interval around the μ value of population change, the standard error around the slopes of linear models of population abundance versus time, and the standard deviation of the raw time-series data for each population. The process noise is a metric of population fluctuations, whereas the rest of the metrics show population variability. The weighted fluctuation models were weighted by τ, the observation error estimate derived from the state-space models.

**Model name**	**Variable**	**Post. mean**	**Lower 95% CI**	**Upper 95% CI**	**Eff. sample size**	**pMCMC**	**Effect**
**Latitude - μ**	(Intercept)	0.003	0.0002	0.005	18,000	0.037	fixed
	Decimal.Latitude	0.00001	-0.00004	0.0001	18,000	0.687	fixed
	sigma	0.003	0.003	0.003	15,774		residual
**Realm - μ**	Freshwater	0.003	-0.001	0.006	18,000	0.146	fixed
	Marine	0.004	0.0003	0.007	18,597	0.029	fixed
	Terrestrial	0.003	0.0002	0.005	18,000	0.035	fixed
	sigma	0.003	0.003	0.003	16,324		residual
**Realm - weighted**	Freshwater	0.004	-0.001	0.009	18,000	0.156	fixed
	Marine	0.003	-0.002	0.008	18,000	0.168	fixed
	Terrestrial	0.003	0.00001	0.007	18,000	0.053	fixed
	sigma	0.003	0.003	0.003	13,851		residual
**Realm - slope**	Freshwater	0.006	-0.0002	0.012	18,000	0.067	fixed
	Marine	0.003	-0.002	0.009	18,000	0.263	fixed
	Terrestrial	0.004	-0.001	0.008	18,000	0.114	fixed
	sigma	0.008	0.008	0.008	15,950		residual
**Realm - fluctuations σ**	Terrestrial	0.022	0.020	0.024	16,584	0.0001	fixed
	Marine	0.028	0.026	0.030	21,131	0.0001	fixed
	Freshwater	0.028	0.025	0.030	17,579	0.0001	fixed

	sigma	0.002	0.002	0.002	15,086		residual
**Realm - fluctuations CI**	Freshwater	0.144	0.136	0.152	18,000	0.0001	fixed
	Marine	0.148	0.140	0.155	18,000	0.0001	fixed
	Terrestrial	0.118	0.111	0.124	18,000	0.0001	fixed
	sigma	0.012	0.011	0.012	17,555		residual
**Realm - fluctuations CI weighted**	Terrestrial	0.130	0.122	0.138	18,000	0.0001	fixed
	Marine	0.169	0.159	0.180	18,000	0.0001	fixed
	Freshwater	0.170	0.159	0.181	16,896	0.0001	fixed
	sigma	0.011	0.010	0.011	18,000		residual
**Realm - fluctuations SE**	Freshwater	0.135	0.126	0.143	18,000	0.0001	fixed
	Marine	0.139	0.131	0.148	18,000	0.0001	fixed
	Terrestrial	0.109	0.102	0.115	18,440	0.0001	fixed
	sigma	0.012	0.011	0.012	18,000		residual
**Realm - fluctuations SD**	Freshwater	0.563	0.557	0.569	18,000	0.0001	fixed
	Marine	0.568	0.562	0.573	18,000	0.0001	fixed
	Terrestrial	0.547	0.542	0.552	18,000	0.0001	fixed
	sigma	0.008	0.008	0.008	16,850		residual
**Biome - μ**	Boreal forests/taiga	0.002	-0.002	0.006	18,664	0.235	fixed
	Deserts and xeric shrublands	-0.006	-0.023	0.012	18,000	0.526	fixed
	Trop. and subtrop. grasslands savannas and shrublands	-0.009	-0.019	0.0004	18,000	0.061	fixed
	Large lakes	0.003	-0.005	0.012	18,000	0.405	fixed
	Mediterranean forests woodlands and scrub	0.005	-0.003	0.014	18,000	0.188	fixed
	Montane freshwaters	0.011	-0.028	0.051	18,000	0.574	fixed
	Montane grasslands and shrublands	0.030	0.010	0.050	18,000	0.003	fixed
	Polar freshwaters	0.004	-0.003	0.012	18,000	0.258	fixed

	Polar seas	-0.011	-0.035	0.014	18,000	0.397	fixed
	Temperate forests	0.002	-0.002	0.005	18,000	0.318	fixed
	Temperate wetlands and rivers	0.002	-0.002	0.007	18,000	0.226	fixed
	Temperate grasslands savannas and shrublands	-0.004	-0.011	0.003	18,000	0.247	fixed
	Tropical wetlands and rivers	0.002	-0.007	0.011	18,000	0.725	fixed
	Tropical and subtropical forests	0.018	0.009	0.027	18,000	0.0001	fixed
	Tropical coral	0.024	0.012	0.037	18,000	0.0001	fixed
	Tundra	0.007	-0.002	0.015	19,239	0.111	fixed
	Xeric freshwaters and endorheic basins	-0.002	-0.017	0.013	18,000	0.831	fixed
	sigma	0.003	0.003	0.003	15,790		residual
**Biome - slope**	Boreal forests/taiga	0.002	-0.004	0.007	18,000	0.523	fixed
	Deserts and xeric shrublands	-0.027	-0.050	-0.002	18,000	0.031	fixed
	Trop. and subtrop. grasslands savannas and shrublands	-0.017	-0.030	-0.004	18,000	0.014	fixed
	Large lakes	0.002	-0.010	0.015	18,781	0.792	fixed
	Mediterranean forests woodlands and scrub	0.010	-0.002	0.023	17,610	0.109	fixed
	Montane freshwaters	0.021	-0.034	0.074	18,000	0.445	fixed
	Montane grasslands and shrublands	0.021	-0.005	0.046	17,224	0.115	fixed
	Polar freshwaters	0.003	-0.009	0.014	18,000	0.660	fixed
	Polar seas	-0.016	-0.046	0.015	17,196	0.320	fixed

	Temperate forests	0.003	-0.001	0.008	17,408	0.165	fixed
	Temperate wetlands and rivers	0.004	-0.001	0.010	18,000	0.123	fixed
	Temperate grasslands savannas and shrublands	-0.001	-0.010	0.007	18,000	0.780	fixed
	Tropical wetlands and rivers	0.003	-0.011	0.016	18,000	0.713	fixed
	Tropical and subtropical forests	0.025	0.012	0.038	17,560	0.0002	fixed
	Tropical coral	0.036	0.019	0.052	18,000	0.0001	fixed
	Tundra	0.013	-0.0003	0.026	17,563	0.048	fixed
	Xeric freshwaters and endorheic basins	0.014	-0.010	0.038	18,257	0.265	fixed
	sigma	0.003	0.003	0.003	13,753		residual
**Biome - weighted**	Boreal forests/taiga	0.003	-0.003	0.010	18,000	0.334	fixed
	Deserts and xeric shrublands	-0.019	-0.049	0.011	18,000	0.208	fixed
	Trop. and subtrop. grasslands savannas and shrublands	-0.004	-0.021	0.013	18,000	0.635	fixed
	Large lakes	0.007	-0.007	0.021	18,000	0.336	fixed
	Mediterranean forests woodlands and scrub	0.006	-0.008	0.020	19,170	0.402	fixed
	Montane freshwaters	0.023	-0.044	0.094	18,000	0.521	fixed
	Montane grasslands and shrublands	0.037	0.003	0.071	20,405	0.033	fixed
	Polar freshwaters	0.012	-0.001	0.025	18,000	0.069	fixed
	Polar seas	-0.023	-0.066	0.018	18,000	0.292	fixed
	Temperate forests	0.003	-0.003	0.009	18,000	0.265	fixed

	Temperate wetlands and rivers	0.005	-0.002	0.012	17,585	0.169	fixed
	Temperate grasslands savannas and shrublands	-0.008	-0.019	0.004	18,000	0.161	fixed
	Tropical wetlands and rivers	0.006	-0.010	0.022	18,000	0.473	fixed
	Tropical and subtropical forests	0.014	-0.001	0.029	18,000	0.068	fixed
	Tropical coral	0.039	0.018	0.060	18,000	0.0003	fixed
	Tundra	0.013	-0.001	0.027	18,000	0.071	fixed
	Xeric freshwaters and endorheic basins	-0.021	-0.048	0.003	18,000	0.099	fixed
	sigma	0.008	0.007	0.008	16,533		residual
**Biome - fluctuations *σ***	Boreal forests/taiga	0.018	0.015	0.021	18,000	0.0001	fixed
	Deserts and xeric shrublands	0.044	0.031	0.057	18,000	0.0001	fixed
	Trop. and subtrop. grasslands savannas and shrublands	0.044	0.037	0.051	18,000	0.0001	fixed
	Large lakes	0.024	0.018	0.030	18,000	0.0001	fixed
	Mediterranean forests woodlands and scrub	0.022	0.016	0.028	18,000	0.0001	fixed
	Montane freshwaters	0.047	0.018	0.075	18,000	0.001	fixed
	Montane grasslands and shrublands	0.031	0.016	0.046	18,000	0.0001	fixed
	Polar freshwaters	0.027	0.021	0.033	17,652	0.0001	fixed
	Polar seas	0.037	0.019	0.055	17,689	0.0001	fixed
	Temperate forests	0.019	0.017	0.022	17,789	0.0001	fixed

	Temperate wetlands and rivers	0.024	0.022	0.027	18,000	0.0001	fixed
	Temperate grasslands savannas and shrublands	0.015	0.010	0.020	18,000	0.0001	fixed
	Tropical wetlands and rivers	0.046	0.039	0.054	18,000	0.0001	fixed
	Tropical and subtropical forests	0.041	0.035	0.048	17,508	0.0001	fixed
	Tropical coral	0.029	0.020	0.038	18,000	0.0001	fixed
	Tundra	0.020	0.014	0.026	18,497	0.0001	fixed
	Xeric freshwaters and endorheic basins	0.039	0.028	0.052	18,845	0.0001	fixed
	sigma	0.002	0.002	0.002	14,440		residual
**Biome - fluctuations CI**	Boreal forests/taiga	0.091	0.083	0.100	18,000	0.0001	fixed
	Deserts and xeric shrublands	0.210	0.173	0.247	17,389	0.0001	fixed
	Trop. and subtrop. grasslands savannas and shrublands	0.200	0.178	0.222	18,000	0.0001	fixed
	Large lakes	0.112	0.095	0.130	18,000	0.0001	fixed
	Mediterranean forests woodlands and scrub	0.132	0.115	0.150	18,000	0.0001	fixed
	Montane freshwaters	0.197	0.108	0.286	18,000	0.0001	fixed
	Montane grasslands and shrublands	0.163	0.121	0.206	18,000	0.0001	fixed
	Polar freshwaters	0.147	0.132	0.164	18,000	0.0001	fixed
	Polar seas	0.146	0.096	0.204	17,894	0.0001	fixed
	Temperate forests	0.099	0.091	0.107	18,000	0.0001	fixed

	Temperate wetlands and rivers	0.132	0.123	0.141	18,000	0.0001	fixed
	Temperate grasslands savannas and shrublands	0.092	0.077	0.106	18,000	0.0001	fixed
	Tropical wetlands and rivers	0.219	0.198	0.239	18,000	0.0001	fixed
	Tropical and subtropical forests	0.197	0.178	0.215	18,405	0.0001	fixed
	Tropical coral	0.161	0.135	0.189	18,000	0.0001	fixed
	Tundra	0.107	0.090	0.124	18,338	0.0001	fixed
	Xeric freshwaters and endorheic basins	0.177	0.146	0.211	18,000	0.0001	fixed
	sigma	0.011	0.011	0.011	17,482		residual
**Biome - fluctuations CI weighted**	Boreal forests/taiga	0.104	0.092	0.115	18,000	0.0001	fixed
	Deserts and xeric shrublands	0.239	0.190	0.288	18,524	0.0001	fixed
	Trop. and subtrop. grasslands savannas and shrublands	0.248	0.220	0.274	18,431	0.0001	fixed
	Large lakes	0.134	0.111	0.158	16,663	0.0001	fixed
	Mediterranean forests woodlands and scrub	0.162	0.137	0.187	18,000	0.0001	fixed
	Montane freshwaters	0.276	0.161	0.393	18,000	0.0001	fixed
	Montane grasslands and shrublands	0.173	0.121	0.222	17,341	0.0001	fixed
	Polar freshwaters	0.172	0.149	0.193	18,386	0.0001	fixed
	Polar seas	0.169	0.103	0.237	18,368	0.0001	fixed
	Temperate forests	0.107	0.097	0.116	18,000	0.0001	fixed

	Temperate wetlands and rivers	0.156	0.144	0.169	18,000	0.0001	fixed
	Temperate grasslands savannas and shrublands	0.107	0.090	0.124	18,000	0.0001	fixed
	Tropical wetlands and rivers	0.239	0.211	0.267	18,000	0.0001	fixed
	Tropical and subtropical forests	0.216	0.190	0.242	18,000	0.0001	fixed
	Tropical coral	0.183	0.147	0.218	18,000	0.0001	fixed
	Tundra	0.114	0.091	0.137	18,000	0.0001	fixed
	Xeric freshwaters and endorheic basins	0.230	0.186	0.273	18,000	0.0001	fixed
	sigma	0.010	0.010	0.011	16,732		residual
**Biome - fluctuations SE**	Boreal forests/taiga	0.086	0.077	0.096	18,000	0.0001	fixed
	Deserts and xeric shrublands	0.204	0.162	0.242	18,000	0.0001	fixed
	Trop. and subtrop. grasslands savannas and shrublands	0.126	0.102	0.150	18,000	0.0001	fixed
	Large lakes	0.095	0.076	0.114	17,101	0.0001	fixed
	Mediterranean forests woodlands and scrub	0.149	0.131	0.167	17,283	0.0001	fixed
	Montane freshwaters	0.184	0.091	0.283	18,000	0.0002	fixed
	Montane grasslands and shrublands	0.110	0.065	0.156	18,000	0.0001	fixed
	Polar freshwaters	0.152	0.134	0.169	18,000	0.0001	fixed
	Polar seas	0.076	0.019	0.137	18,000	0.011	fixed
	Temperate forests	0.094	0.086	0.103	18,000	0.0001	fixed

	Temperate wetlands and rivers	0.123	0.113	0.133	18,000	0.0001	fixed
	Temperate grasslands savannas and shrublands	0.096	0.080	0.111	18,000	0.0001	fixed
	Tropical wetlands and rivers	0.229	0.207	0.251	18,000	0.0001	fixed
	Tropical and subtropical forests	0.154	0.133	0.174	18,000	0.0001	fixed
	Tropical coral	0.187	0.158	0.218	18,580	0.0001	fixed
	Tundra	0.111	0.092	0.129	18,663	0.0001	fixed
	Xeric freshwaters and endorheic basins	0.129	0.095	0.163	18,000	0.0001	fixed
	sigma	0.012	0.011	0.012	17,110		residual
**Biome - fluctuations SD**	Boreal forests/taiga	0.523	0.517	0.530	18,000	0.0001	fixed
	Deserts and xeric shrublands	0.585	0.556	0.614	18,000	0.0001	fixed
	Trop. and subtrop. grasslands savannas and shrublands	0.602	0.585	0.619	18,000	0.0001	fixed
	Large lakes	0.540	0.527	0.555	18,000	0.0001	fixed
	Mediterranean forests woodlands and scrub	0.574	0.560	0.587	18,300	0.0001	fixed
	Montane freshwaters	0.605	0.537	0.671	18,000	0.0001	fixed
	Montane grasslands and shrublands	0.583	0.549	0.616	18,000	0.0001	fixed
	Polar freshwaters	0.570	0.557	0.582	18,000	0.0001	fixed
	Polar seas	0.548	0.504	0.588	18,000	0.0001	fixed
	Temperate forests	0.536	0.531	0.542	18,000	0.0001	fixed

	Temperate wetlands and rivers	0.557	0.551	0.564	18,000	0.0001	fixed
	Temperate grasslands savannas and shrublands	0.526	0.515	0.537	18,000	0.0001	fixed
	Tropical wetlands and rivers	0.608	0.593	0.625	18,000	0.0001	fixed
	Tropical and subtropical forests	0.614	0.600	0.629	18,000	0.0001	fixed
	Tropical coral	0.609	0.588	0.629	18,264	0.0001	fixed
	Tundra	0.539	0.525	0.553	18,000	0.0001	fixed
	Xeric freshwaters and endorheic basins	0.556	0.530	0.580	18,000	0.0001	fixed
	sigma	0.007	0.007	0.008	17,425		residual
**Taxa - *μ***	Actinopterygii	0.00004	-0.004	0.004	18,851	0.986	fixed
	Amphibia	-0.012	-0.022	-0.002	17,369	0.027	fixed
	Aves	0.003	0.001	0.006	18,000	0.007	fixed
	Elasmobranchii	-0.010	-0.022	0.002	17,095	0.097	fixed
	Mammalia	0.010	0.005	0.015	19,689	0.0001	fixed
	Reptilia	0.010	0.001	0.020	18,000	0.029	fixed
	sigma	0.003	0.003	0.003	16,346		residual
**Taxa - weighted**	Actinopterygii	-0.001	-0.007	0.004	18,000	0.744	fixed
	Amphibia	-0.016	-0.032	-0.001	18,000	0.046	fixed
	Aves	0.005	0.002	0.008	18,000	0.003	fixed
	Elasmobranchii	-0.017	-0.035	0.002	18,000	0.077	fixed
	Mammalia	0.011	0.004	0.017	18,490	0.001	fixed
	Reptilia	0.004	-0.010	0.018	18,000	0.578	fixed
	sigma	0.003	0.003	0.003	14,129		residual
**Taxa - slope**	Actinopterygii	-0.001	-0.008	0.005	18,000	0.664	fixed
	Amphibia	-0.020	-0.037	-0.002	16,874	0.026	fixed
	Aves	0.006	0.001	0.010	18,000	0.009	fixed
	Elasmobranchii	-0.018	-0.039	0.002	17,613	0.083	fixed
	Mammalia	0.011	0.003	0.020	18,000	0.012	fixed
	Reptilia	0.022	0.006	0.038	18,000	0.007	fixed
	sigma	0.008	0.008	0.008	16,177		residual

**Taxa - fluctuations σ**	Actinopterygii	0.032	0.030	0.035	17,489	0.0001	fixed
	Amphibia	0.040	0.033	0.047	18,000	0.0001	fixed
	Aves	0.018	0.017	0.020	18,000	0.0001	fixed
	Elasmobranchii	0.030	0.022	0.039	18,000	0.0001	fixed
	Mammalia	0.035	0.032	0.038	17,868	0.0001	fixed
	Reptilia	0.034	0.028	0.041	18,000	0.0001	fixed
	sigma	0.002	0.002	0.002	13,289		residual
**Taxa - fluctuations CI**	Actinopterygii	0.163	0.155	0.171	18,000	0.0001	fixed
	Amphibia	0.208	0.186	0.231	18,000	0.0001	fixed
	Aves	0.094	0.089	0.099	17,127	0.0001	fixed
	Elasmobranchii	0.152	0.125	0.177	18,000	0.0001	fixed
	Mammalia	0.182	0.172	0.193	18,000	0.0001	fixed
	Reptilia	0.195	0.176	0.216	18,476	0.0001	fixed
	sigma	0.012	0.011	0.012	18,000		residual
**Taxa - fluctuations CI weighted**	Actinopterygii	0.191	0.180	0.202	18,000	0.0001	fixed
	Amphibia	0.272	0.242	0.304	17,535	0.0001	fixed
	Aves	0.105	0.098	0.112	18,000	0.0001	fixed
	Elasmobranchii	0.197	0.160	0.232	18,434	0.0001	fixed
	Mammalia	0.212	0.198	0.226	19,200	0.0001	fixed
	Reptilia	0.229	0.201	0.257	18,000	0.0001	fixed
	sigma	0.011	0.011	0.012	17,175		residual
**Taxa - fluctuations SE**	Actinopterygii	0.159	0.150	0.168	18,000	0.0001	fixed
	Amphibia	0.224	0.199	0.247	17,493	0.0001	fixed
	Aves	0.080	0.074	0.086	18,000	0.0001	fixed
	Elasmobranchii	0.151	0.124	0.179	18,000	0.0001	fixed
	Mammalia	0.157	0.145	0.169	18,000	0.0001	fixed
	Reptilia	0.233	0.211	0.255	18,000	0.0001	fixed
	sigma	0.011	0.011	0.012	18,000		residual
**Taxa - fluctuations SD**	Actinopterygii	0.572	0.566	0.579	18,000	0.0001	fixed
	Amphibia	0.612	0.596	0.630	18,000	0.0001	fixed
	Aves	0.530	0.526	0.534	18,000	0.0001	fixed
	Elasmobranchii	0.563	0.542	0.582	18,000	0.0001	fixed
	Mammalia	0.603	0.595	0.611	18,000	0.0001	fixed
	Reptilia	0.610	0.595	0.625	18,000	0.0001	fixed
	sigma	0.008	0.007	0.008	18,000		residual

**Number of time points - *μ***	points	0.00002	-0.00004	0.0001	18,000	0.464	fixed
	sigma	0.003	0.003	0.003	16,697		residual
**Number of time points – *σ* (fluctuations)**	points	0.0002	0.0001	0.0003	16,224	0.0001	fixed
	sigma	0.002	0.002	0.002	16,816		residual
**Duration**	duration	0.0001	0.00003	0.0001	18,398	0.001	fixed
	sigma	0.003	0.003	0.004	18,000		residual
**Duration * System interaction**	Freshwater	0.006	0.002	0.010	17,386	0.008	fixed
	Marine	0.009	0.004	0.014	18,000	0.001	fixed
	Terrestrial	0.009	0.006	0.013	18,000	0.0001	fixed
	duration	-0.0001	-0.0002	0.0001	18,025	0.532	fixed
	Marine:duration	-0.0002	-0.001	0.0001	18,000	0.162	fixed
	Terrestrial:duration	-0.0002	-0.0004	0.00004	17,941	0.113	fixed
	sigma	0.003	0.003	0.004	18,062		residual
**Duration * Taxa interaction**	Actinopterygii	0.0005	-0.006	0.007	18,000	0.890	fixed
	Amphibia	-0.019	-0.039	0.002	18,000	0.077	fixed
	Aves	0.010	0.006	0.014	18,000	0.0001	fixed
	Mammalia	0.011	0.003	0.019	18,000	0.009	fixed
	Reptilia	0.023	0.010	0.038	18,000	0.001	fixed
	duration	-0.00003	-0.0004	0.0003	18,000	0.879	fixed
	Amphibia:duration	0.001	-0.001	0.002	18,000	0.444	fixed
	Aves:duration	-0.0002	-0.001	0.0001	18,000	0.242	fixed
	Mammalia:duration	-0.00001	-0.001	0.001	18,000	0.956	fixed
	Reptilia:duration	-0.001	-0.002	-0.0001	18,000	0.026	fixed
	sigma	0.003	0.003	0.003	16,297		residual
**Sampling units - *μ***	(Intercept)	-0.0001	-0.003	0.003	18,000	0.918	fixed
	Individuals (1167)	0.006	0.002	0.010	18,000	0.003	fixed
	Pairs (505)	0.015	0.009	0.020	18,000	0.0001	fixed
	Nests (224)	0.015	0.007	0.023	18,000	0.0002	fixed
	Population estimate (97)	0.017	0.007	0.027	18,000	0.001	fixed
	sigma	0.002	0.002	0.002	15,983		residual

**Geographic range birds/mammals - *μ***	(Intercept)	0.012	-0.002	0.026	18,000	0.082	fixed
	log(Geographic range)	-0.0005	-0.001	0.0004	18,000	0.278	fixed
	sigma	0.003	0.003	0.003	17,575		residual
**Geographic range birds/mammals - *μ* * taxa interaction**	(Intercept)	0.068	0.031	0.107	18,000	0.0002	fixed
	log(Geographic range)	-0.004	-0.007	-0.002	18,000	0.001	fixed
	Aves	-0.076	-0.119	-0.031	18,000	0.001	fixed
	log(Geographic range):Aves	0.005	0.002	0.008	18,000	0.001	fixed
	sigma	0.003	0.003	0.003	16,888		residual
**Geographic range birds/mammals - weighted**	(Intercept)	0.013	-0.011	0.038	18,000	0.295	fixed
	log(Geographic range)	-0.0004	-0.002	0.001	18,000	0.564	fixed
	sigma	0.003	0.003	0.003	16,791		residual
**Geographic range birds/mammals - slope**	(Intercept)	0.010	-0.020	0.041	18,433	0.515	fixed
	log.range	-0.0002	-0.002	0.002	18,408	0.813	fixed
	sigma	0.008	0.007	0.008	16,986		residual
**Geographic range birds/mammals - fluctuations *σ***	(Intercept)	0.046	0.032	0.060	18,000	0.0001	fixed
	log(Geographic range)	-0.001	-0.002	-0.001	18,000	0.001	fixed
	sigma	0.002	0.002	0.002	15,577		residual
**Geographic range birds/mammals - fluctuations CI**	(Intercept)	0.180	0.137	0.226	18,000	0.0001	fixed
	log(Geographic range)	-0.004	-0.007	-0.001	18,769	0.003	fixed
	sigma	0.011	0.011	0.011	18,000		residual

**Geographic range birds/mammals - fluctuations CI weighted**	(Intercept)	0.173	0.117	0.233	18,000	0.0001	fixed
	log(Geographic range)	-0.003	-0.006	0.001	18,000	0.122	fixed
	sigma	0.011	0.010	0.011	18,000		residual
**Geographic range birds/mammals - fluctuations SE**	(Intercept)	0.082	0.037	0.126	17,605	0.0001	fixed
	log(Geographic range)	0.001	-0.002	0.004	17,534	0.512	fixed
	sigma	0.011	0.011	0.011	18,000		residual
**Geographic range birds/mammals - fluctuations SD**	(Intercept)	0.605	0.571	0.638	17,938	0.0001	fixed
	log(Geographic range)	-0.004	-0.006	-0.002	17,306	0.0004	fixed
	sigma	0.008	0.007	0.008	18,000		residual
**Mean population size - *μ***	(Intercept)	0.001	-0.003	0.005	18,000	0.794	fixed
	log(Mean population size)	0.001	0.001	0.002	18,000	0.0004	fixed
	sigma	0.004	0.004	0.004	15,837		residual
**Mean population size - *μ* * taxa interaction**	(Intercept)	0.007	-0.003	0.018	18,000	0.161	fixed
	log(Mean population size)	-0.001	-0.003	0.001	18,000	0.197	fixed
	Amphibia	-0.006	-0.027	0.017	18,000	0.615	fixed
	Aves	-0.009	-0.021	0.003	18,000	0.133	fixed
	Elasmobranchii	-0.025	-0.050	0.0004	18,000	0.055	fixed
	Mammalia	-0.009	-0.022	0.006	18,000	0.211	fixed
	Reptilia	0.007	-0.011	0.025	18,000	0.434	fixed
	log(Mean population size):Amphibia	-0.003	-0.008	0.002	19,145	0.215	fixed

	log(Mean population size):Aves	0.003	0.001	0.005	18,000	0.003	fixed
	log(Mean population size):Elasmobranchii	0.005	-0.001	0.012	18,428	0.127	fixed
	log(Mean population size):Mammalia	0.004	0.001	0.006	18,000	0.002	fixed
	log(Mean population size):Reptilia	0.001	-0.003	0.004	18,000	0.772	fixed
	sigma	0.004	0.004	0.004	16,486		residual
**Mean population size - weighted**	(Intercept)	0.002	-0.005	0.008	18,000	0.611	fixed
	log(Mean population size)	0.001	0.0003	0.002	18,000	0.014	fixed
	sigma	0.005	0.004	0.005	13,784		residual
**Mean population size - slope**	(Intercept)	-0.0002	-0.007	0.006	18,000	0.946	fixed
	log(Mean population size)	0.002	0.001	0.003	18,000	0.0002	fixed
	sigma	0.011	0.010	0.012	15,825		residual
**Mean population size - fluctuations *σ***	(Intercept)	0.034	0.032	0.037	18,000	0.0001	fixed
	log(Mean population size)	-0.001	-0.001	-0.0002	18,000	0.003	fixed
	sigma	0.002	0.002	0.002	6,763		residual
**Mean population size - fluctuations CI**	(Intercept)	0.222	0.212	0.232	18,000	0.0001	fixed
	log(Mean population size)	-0.004	-0.006	-0.003	18,000	0.0001	fixed
	sigma	0.026	0.025	0.027	14,178		residual
**Mean population size - fluctuations CI weighted**	(Intercept)	0.262	0.249	0.275	18,000	0.0001	fixed

	log(Mean population size)	-0.007	-0.009	-0.004	18,000	0.0001	fixed
	sigma	0.026	0.025	0.028	13,162		residual
**Mean population size - fluctuations SE**	(Intercept)	0.228	0.218	0.238	18,000	0.0001	fixed
	log(Mean population size)	-0.010	-0.012	-0.008	18,000	0.0001	fixed
	sigma	0.023	0.022	0.024	15,480		residual
**Mean population size - fluctuations SD**	(Intercept)	0.001	-0.001	0.003	17,324	0.454	fixed
	log(Mean population size)	0.00000	-0.00001	0.00002	18,000	0.556	fixed
	sigma	0.00000	0.00000	0.00000	18,000		residual
**Habitat specificity - *μ***	(Intercept)	0.002	-0.001	0.006	18,000	0.176	fixed
	Habitat specificity	0.0002	-0.0002	0.001	18,000	0.321	fixed
	sigma	0.003	0.003	0.003	15,647		residual
**Habitat specificity - *μ* * taxa interaction**	(Intercept)	0.003	-0.004	0.011	18,000	0.443	fixed
	Habitat specificity	-0.0004	-0.002	0.001	18,187	0.538	fixed
	Amphibia	-0.015	-0.035	0.004	17,354	0.130	fixed
	Aves	-0.002	-0.011	0.007	18,000	0.652	fixed
	Cephalaspidomorphi	0.016	-0.103	0.132	18,000	0.796	fixed
	Elasmobranchii	-0.027	-0.055	0.001	17,291	0.059	fixed
	Holocephali	-0.105	-0.224	0.013	18,000	0.082	fixed
	Mammalia	0.008	-0.003	0.018	18,000	0.168	fixed
	Myxini	-0.063	-0.145	0.022	18,000	0.146	fixed
	Reptilia	0.015	-0.007	0.036	18,000	0.176	fixed
	Habitat specificity: Amphibia	0.0003	-0.002	0.002	17,509	0.753	fixed
	Habitat specificity: Aves	0.001	-0.001	0.002	18,000	0.261	fixed
	Habitat specificity:	0.005	-0.003	0.013	18,000	0.264	fixed

	Elasmobranchii						
	Habitat specificity: Mammalia	0.001	-0.001	0.002	19,769	0.494	fixed
	Habitat specificity: Reptilia	-0.001	-0.004	0.002	18,000	0.503	fixed
	sigma	0.003	0.003	0.003	16,057		residual
**Habitat specificity - weighted**	(Intercept)	0.002	-0.003	0.006	18,000	0.443	fixed
	Habitat specificity	0.0004	-0.0001	0.001	18,000	0.128	fixed
	sigma	0.003	0.003	0.003	13,856		residual
**Habitat specificity - slope**	(Intercept)	0.003	-0.003	0.009	18,000	0.317	fixed
	Habitat specificity	0.0003	-0.0003	0.001	18,000	0.321	fixed
	sigma	0.008	0.008	0.009	16,422		residual
**Habitat specificity - fluctuations *σ***	(Intercept)	0.023	0.021	0.025	16,596	0.0001	fixed
	Habitat specificity	0.0002	-0.0001	0.0005	17,782	0.123	fixed
	sigma	0.002	0.002	0.002	14,047		residual
**Habitat specificity - fluctuations CI**	(Intercept)	0.126	0.118	0.134	18,000	0.0001	fixed
	Habitat specificity	0.001	-0.0002	0.002	18,000	0.117	fixed
	sigma	0.012	0.011	0.012	18,411		residual
**Habitat specificity - fluctuations CI weighted**	(Intercept)	0.140	0.129	0.151	18,000	0.0001	fixed
	Habitat specificity	0.001	-0.0001	0.002	18,000	0.065	fixed
	sigma	0.011	0.011	0.012	18,131		residual
**Habitat specificity - fluctuations SE**	(Intercept)	0.117	0.108	0.126	18,000	0.0001	fixed
	Habitat specificity	0.001	-0.0002	0.002	18,000	0.138	fixed
	sigma	0.012	0.012	0.012	17,484		residual
**Habitat specificity**	(Intercept)	0.555	0.549	0.561	18,000	0.0001	fixed

**- fluctuations SD**							
	Habitat specificity	0.0003	-0.0004	0.001	16,414	0.349	fixed
	sigma	0.008	0.008	0.008	18,000		residual
**IUCN Red List Categories - *μ***	Least concern	0.005	0.003	0.008	18,000	0.0001	fixed
	Near threatened	-0.004	-0.012	0.004	18,000	0.319	fixed
	Vulnerable	0.003	-0.005	0.010	18,000	0.427	fixed
	Endangered	-0.004	-0.013	0.006	18,000	0.452	fixed
	Critically endangered	-0.007	-0.020	0.005	18,541	0.259	fixed
	sigma	0.003	0.003	0.003	15,497		residual
**IUCN Red List Categories - weighted**	Least concern	0.007	0.004	0.010	18,000	0.0001	fixed
	Near threatened	0.001	-0.010	0.011	18,241	0.852	fixed
	Vulnerable	0.003	-0.008	0.013	18,000	0.626	fixed
	Endangered	-0.007	-0.020	0.006	18,000	0.291	fixed
	Critically endangered	-0.014	-0.032	0.004	18,000	0.114	fixed
	sigma	0.003	0.003	0.003	14,285		residual
**IUCN Red List Categories - slope**	Least concern	0.009	0.005	0.012	18,905	0.0001	fixed
	Near threatened	-0.011	-0.024	0.003	18,000	0.121	fixed
	Vulnerable	0.003	-0.010	0.016	16,528	0.684	fixed
	Endangered	-0.007	-0.023	0.009	18,000	0.423	fixed
	Critically endangered	-0.013	-0.034	0.009	18,000	0.254	fixed
	sigma	0.008	0.008	0.009	15,931		residual
**IUCN Red List Categories - fluctuations *σ***	Least concern	0.023	0.022	0.025	16,756	0.0001	fixed
	Near threatened	0.028	0.023	0.033	17,565	0.0001	fixed
	Vulnerable	0.027	0.022	0.032	20,081	0.0001	fixed

	Endangered	0.032	0.026	0.039	18,000	0.0001	fixed
	Critically endangered	0.039	0.031	0.048	18,000	0.0001	fixed
	sigma	0.002	0.002	0.002	13,906		residual
**IUCN Red List Categories - fluctuations CI**	Least concern	0.124	0.118	0.129	18,000	0.0001	fixed
	Near threatened	0.152	0.134	0.171	18,000	0.0001	fixed
	Vulnerable	0.151	0.134	0.169	17,076	0.0001	fixed
	Endangered	0.168	0.146	0.189	20,476	0.0001	fixed
	Critically endangered	0.178	0.150	0.208	18,000	0.0001	fixed
	sigma	0.012	0.012	0.012	17,734		residual
**IUCN Red List Categories - fluctuations CI weighted**	Least concern	0.140	0.133	0.147	18,000	0.0001	fixed
	Near threatened	0.172	0.147	0.196	17,244	0.0001	fixed
	Vulnerable	0.171	0.148	0.195	18,589	0.0001	fixed
	Endangered	0.194	0.165	0.222	18,000	0.0001	fixed
	Critically endangered	0.193	0.155	0.231	18,000	0.0001	fixed
	sigma	0.012	0.011	0.012	17,086		residual
**IUCN Red List Categories - fluctuations SE**	Least concern	0.119	0.113	0.125	18,000	0.0001	fixed
	Near threatened	0.135	0.114	0.156	18,000	0.0001	fixed
	Vulnerable	0.127	0.107	0.147	18,000	0.0001	fixed
	Endangered	0.143	0.119	0.167	18,000	0.0001	fixed
	Critically endangered	0.149	0.116	0.180	17,444	0.0001	fixed
	sigma	0.012	0.012	0.012	18,000		residual
**IUCN Red List Categories -**	Least concern	0.550	0.546	0.554	18,000	0.0001	fixed

**fluctuations SD**							
	Near threatened	0.575	0.561	0.589	18,000	0.0001	fixed
	Vulnerable	0.573	0.559	0.586	17,618	0.0001	fixed
	Endangered	0.596	0.580	0.613	18,000	0.0001	fixed
	Critically endangered	0.604	0.582	0.625	17,544	0.0001	fixed
	sigma	0.008	0.008	0.008	17,153		residual
**IUCN threat type - *μ***	Fishing / harvesting aquatic resources	0.005	-0.0005	0.010	18,000	0.085	fixed
	Hunting / trapping terrestrial animals	0.005	-0.0003	0.010	18,000	0.068	fixed
	Annual / perennial non-timber crops	0.005	-0.0004	0.012	18,000	0.080	fixed
	Intentional use (species is the target)	0.005	-0.001	0.011	18,000	0.092	fixed
	Habitat shifting / alteration	0.005	-0.001	0.011	18,000	0.098	fixed
	Housing / urban areas	0.004	-0.002	0.011	17,916	0.210	fixed
	Unintentional effects: (large scale) [harvest]	0.004	-0.002	0.010	19,228	0.205	fixed
	Intentional use: (subsistence/small scale) [harvest]	0.005	-0.002	0.012	18,423	0.192	fixed
	Agricultural / forestry effluents	0.005	-0.002	0.011	18,000	0.153	fixed
	Intentional use: (large scale) [harvest]	0.004	-0.003	0.011	17,575	0.257	fixed
	sigma	0.004	0.004	0.004	18,000		residual
**IUCN threat number - *μ***	(Intercept)	0.004	-0.001	0.009	18,000	0.145	fixed
	Number of threats	0.0001	-0.0004	0.001	18,000	0.726	fixed

	sigma	0.004	0.004	0.004	16,084		residual
**IUCN threat type - fluctuations *σ***	Fishing / harvesting aquatic resources	0.033	0.028	0.037	17,468	0.0001	fixed
	Hunting / trapping terrestrial animals	0.033	0.029	0.038	17,547	0.0001	fixed
	Annual / perennial non-timber crops	0.033	0.029	0.038	17,225	0.0001	fixed
	Intentional use (species is the target)	0.033	0.029	0.038	18,000	0.0001	fixed
	Habitat shifting / alteration	0.032	0.028	0.037	17,548	0.0001	fixed
	Housing / urban areas	0.034	0.029	0.039	18,000	0.0001	fixed
	Unintentional effects: (large scale) [harvest]	0.033	0.028	0.037	17,134	0.0001	fixed
	Intentional use: (subsistence/small scale) [harvest]	0.033	0.028	0.038	18,000	0.0001	fixed
	Agricultural / forestry effluents	0.033	0.028	0.037	17,571	0.0001	fixed
	Intentional use: (large scale) [harvest]	0.032	0.027	0.037	18,000	0.0001	fixed
	sigma	0.002	0.002	0.002	18,000		residual
**IUCN threat number - fluctuations *σ***	(Intercept)	0.029	0.026	0.032	17,078	0.0001	fixed
	Number of threats	0.0001	-0.0002	0.0004	18,000	0.475	fixed
	sigma	0.002	0.002	0.002	7,021		residual

Supplementary Table 3. Model outputs from UK-scale analyses. Sigma is the overall model residual variance. Net population change is estimated using μ values derived from state-space models of population abundance versus time and slopes of linear models of population abundance versus time. The weighted μ models included μ as a response variable, weighted by τ, the observation error estimate derived from the state-space models. The fluctuation models included the process noise (σ^2) values from state-space models, half of the 95% confidence interval around the μ value of population change, the standard error around the slopes of linear models of population abundance versus time, and the standard deviation of the raw time-series data for each population. The process noise is a metric of population fluctuations, whereas the rest of the metrics show population variability. The weighted fluctuation models were weighted by τ, the observation error estimate derived from the state-space models.

Model name	Variable	Posterior mean	Lower 95% CI	Upper 95% CI	Effective sample size	pMCMC	Effect
**Realm - μ**	Terrestrial	0.011	-0.003	0.023	18,000	0.105	fixed
	Marine	0.007	-0.006	0.020	18,000	0.297	fixed
	Freshwater	0.030	0.010	0.049	17,098	0.002	fixed
	sigma	0.003	0.003	0.004	13,565		residual
**Realm - weighted**	Terrestrial	0.015	0.0004	0.030	18,000	0.038	fixed
	Marine	0.008	-0.008	0.024	18,000	0.303	fixed
	Freshwater	0.027	0.005	0.050	18,000	0.021	fixed
	sigma	0.003	0.002	0.004	10,568		residual
**Realm - slope**	Freshwater	0.062	0.025	0.098	18,000	0.0003	fixed
	Marine	0.007	-0.018	0.031	18,000	0.601	fixed
	Terrestrial	0.020	-0.004	0.045	18,380	0.114	fixed
	sigma	0.011	0.009	0.012	15,525		residual
**Realm - fluctuations σ**	Terrestrial	0.085	0.062	0.105	18,000	0.0001	fixed
	Marine	0.056	0.034	0.079	18,000	0.0001	fixed
	Freshwater	0.051	0.019	0.083	18,000	0.002	fixed
	sigma	0.005	0.004	0.006	16,954		residual
**Realm - fluctuations CI**	Terrestrial	0.193	0.165	0.220	18,000	0.0001	fixed
	Marine	0.118	0.090	0.146	18,000	0.0001	fixed
	Freshwater	0.139	0.100	0.181	18,000	0.0001	fixed
	sigma	0.011	0.009	0.013	15,777		residual

**Realm - fluctuations CI weighted**	Terrestrial	0.191	0.157	0.224	18,000	0.0001	fixed
	Marine	0.134	0.098	0.172	18,000	0.0001	fixed
	Freshwater	0.164	0.115	0.217	18,001	0.0001	fixed
	sigma	0.010	0.007	0.012	15,014		residual
**Taxa - μ**	Actinopterygii	-0.003	-0.020	0.014	16,879	0.744	fixed
	Amphibia	-0.0002	-0.051	0.052	18,000	0.995	fixed
	Aves	0.012	0.001	0.022	18,000	0.027	fixed
	Elasmobranchii	0.006	-0.045	0.056	17,998	0.811	fixed
	Mammalia	0.046	0.023	0.069	17,742	0.0001	fixed
	Reptilia	0.044	-0.018	0.108	18,000	0.181	fixed
	sigma	0.004	0.003	0.004	10,343		residual
**Taxa - weighted**	Actinopterygii	-0.010	-0.033	0.011	18,000	0.374	fixed
	Amphibia	-0.002	-0.065	0.061	18,000	0.947	fixed
	Aves	0.017	0.005	0.029	18,000	0.005	fixed
	Elasmobranchii	0.002	-0.089	0.086	18,000	0.972	fixed
	Mammalia	0.043	0.015	0.069	18,000	0.002	fixed
	Reptilia	0.030	-0.039	0.103	18,000	0.404	fixed
	sigma	0.003	0.002	0.004	9,676		residual
**Taxa - slope**	Actinopterygii	-0.009	-0.042	0.024	17,082	0.593	fixed
	Amphibia	0.004	-0.095	0.111	18,000	0.931	fixed
	Aves	0.020	0.0001	0.040	18,434	0.046	fixed
	Elasmobranchii	-0.014	-0.106	0.078	18,000	0.766	fixed
	Mammalia	0.099	0.053	0.145	18,000	0.0001	fixed
	Reptilia	0.069	-0.048	0.185	19,449	0.241	fixed
	sigma	0.011	0.009	0.013	14,664		residual
**Taxa - fluctuations σ**	Actinopterygii	0.051	0.019	0.082	18,000	0.001	fixed
	Amphibia	0.083	-0.023	0.182	18,000	0.117	fixed
	Aves	0.073	0.053	0.091	18,000	0.0001	fixed
	Elasmobranchii	0.072	-0.010	0.154	18,000	0.083	fixed
	Mammalia	0.054	0.012	0.098	18,000	0.014	fixed
	Reptilia	0.123	0.014	0.222	18,000	0.021	fixed
	sigma	0.005	0.004	0.006	17,207		residual
**Taxa - fluctuations CI**	Actinopterygii	0.113	0.074	0.153	18,891	0.0001	fixed
	Amphibia	0.230	0.107	0.358	18,000	0.0004	fixed
	Aves	0.166	0.143	0.190	18,000	0.0001	fixed
	Elasmobranchii	0.186	0.078	0.290	18,000	0.001	fixed
	Mammalia	0.150	0.095	0.204	18,761	0.0001	fixed
	Reptilia	0.275	0.146	0.410	18,000	0.0002	fixed
	sigma	0.011	0.009	0.013	15,974		residual
**Taxa - fluctuations CI weighted**	Actinopterygii	0.138	0.088	0.190	18,000	0.0001	fixed
	Amphibia	0.287	0.134	0.448	18,000	0.001	fixed
	Aves	0.169	0.142	0.197	18,255	0.0001	fixed

	Elasmobranchii	0.266	0.097	0.432	17,623	0.003	fixed
	Mammalia	0.156	0.090	0.222	18,000	0.0001	fixed
	Reptilia	0.311	0.158	0.474	18,000	0.0001	fixed
	sigma	0.009	0.007	0.012	14,900		residual
**Geographic range (all) - *μ***	(Intercept)	0.006	-0.052	0.063	18,000	0.850	fixed
	log(km2_range)	0.001	-0.003	0.005	18,000	0.762	fixed
	sigma	0.004	0.003	0.004	14,513		residual
**Geographic range (all) - weighted**	(Intercept)	-0.012	-0.077	0.050	18,000	0.710	fixed
	log.range	0.002	-0.002	0.006	18,000	0.346	fixed
	sigma	0.003	0.002	0.004	13,236		residual
**Geographic range (all) - slope**	(Intercept)	0.009	-0.105	0.120	18,239	0.870	fixed
	log.range	0.001	-0.006	0.009	18,257	0.755	fixed
	sigma	0.011	0.009	0.013	16,452		residual
**Geographic range (all) - fluctuations *σ***	(Intercept)	0.004	-0.101	0.115	18,000	0.939	fixed
	log(km2_range)	0.005	-0.003	0.012	18,000	0.198	fixed
	sigma	0.005	0.004	0.006	18,000		residual
**Geographic range (all) - fluctuations CI**	(Intercept)	0.045	-0.086	0.177	18,000	0.494	fixed
	log.range	0.008	-0.001	0.017	18,000	0.079	fixed
	sigma	0.011	0.009	0.013	16,713		residual
**Geographic range (all) - fluctuations CI weighted**	(Intercept)	0.059	-0.104	0.221	18,000	0.472	fixed
	log.range	0.008	-0.003	0.019	18,000	0.162	fixed
	sigma	0.010	0.008	0.013	15,715		residual
**Geographic range (all) - fluctuations SE**	(Intercept)	0.050	-0.104	0.206	18,000	0.528	fixed
	log.range	0.008	-0.003	0.018	18,000	0.159	fixed
	sigma	0.009	0.007	0.010	18,000		residual
**Geographic range (all) - fluctuations SD**	(Intercept)	0.171	0.039	0.294	18,000	0.008	fixed
	log.range	0.016	0.007	0.025	18,000	0.0002	fixed
	sigma	0.017	0.014	0.020	15,395		residual
**Mean population size - *μ***	(Intercept)	0.023	0.0001	0.047	1,207	0.051	fixed
	log(Mean population size)	-0.0004	-0.004	0.004	2,374	0.860	fixed
	sigma	0.006	0.005	0.008	411		residual
**Mean population size - weighted**	(Intercept)	0.021	-0.002	0.045	852	0.066	fixed
	log(Mean population size)	-0.0001	-0.004	0.004	1,950	0.964	fixed
	sigma	0.007	0.005	0.009	222		residual

**Mean population size - slope**	(Intercept)	0.035	-0.006	0.075	1,274	0.071	fixed
	log(meanpop)	-0.001	-0.008	0.006	6,788	0.832	fixed
	sigma	0.022	0.016	0.027	593		residual
**Mean population size - fluctuations *σ***	(Intercept)	0.169	0.118	0.216	18,000	0.0001	fixed
	log(Mean population size)	-0.004	-0.013	0.005	18,000	0.389	fixed
	sigma	0.034	0.023	0.045	200		residual
**Mean population size - fluctuations CI**	(Intercept)	0.298	0.242	0.357	18,000	0.0001	fixed
	log(Mean population size)	-0.009	-0.019	0.001	18,000	0.080	fixed
	sigma	0.026	0.018	0.035	11,283		residual
**Mean population size - fluctuations CI weighted**	(Intercept)	0.298	0.240	0.354	18,305	0.0001	fixed
	log(Mean population size)	-0.009	-0.019	0.001	18,000	0.077	fixed
	sigma	0.026	0.018	0.035	10,953		residual
**Mean population size - fluctuations SE**	(Intercept)	0.354	0.293	0.411	18,000	0.0001	fixed
	log(meanpop)	-0.024	-0.034	-0.014	18,099	0.0001	fixed
	sigma	0.014	0.009	0.018	16,950		residual
**Mean population size - fluctuations SD**	(Intercept)	0.519	0.473	0.565	17,529	0.0001	fixed
	log(Mean population size)	-0.007	-0.016	0.001	17,123	0.086	fixed
	sigma	0.030	0.019	0.039	83		residual
**Habitat specificity - *μ***	(Intercept)	0.007	-0.010	0.024	18,000	0.449	fixed
	Habitat specificity	0.001	-0.001	0.002	18,000	0.340	fixed
	sigma	0.004	0.003	0.005	13,436		residual
**Habitat specificity (profiling) - *μ***	(Intercept)	0.013	-0.005	0.030	18,000	0.173	fixed
	Habitat specificity	-0.0002	-0.003	0.002	18,000	0.864	fixed
	sigma	0.005	0.004	0.005	8,201		residual
**Habitat specificity - weighted**	(Intercept)	0.009	-0.012	0.030	18,441	0.381	fixed
	Habitat specificity	0.001	-0.001	0.003	18,000	0.424	fixed
	sigma	0.003	0.002	0.004	10,360		residual
**Habitat specificity - slope**	(Intercept)	0.013	-0.020	0.046	17,956	0.434	fixed
	Habitat specificity	0.001	-0.002	0.004	17,589	0.434	fixed
	sigma	0.011	0.009	0.014	13,969		residual

**Habitat specificity (profiling) - slope**	(Intercept)	0.015	-0.020	0.049	18,509	0.394	fixed
	Habitat specificity	0.0004	-0.004	0.005	17,454	0.852	fixed
	sigma	0.013	0.011	0.016	12,446		residual
**Habitat specificity - fluctuations *σ***	(Intercept)	0.055	0.027	0.085	18,000	0.0003	fixed
	Habitat specificity	0.002	-0.001	0.004	17,598	0.221	fixed
	sigma	0.005	0.004	0.006	17,516		residual
**Habitat specificity (profiling) - fluctuations *σ***	(Intercept)	0.022	0.008	0.038	18,947	0.004	fixed
	Habitat specificity	0.002	-0.0003	0.004	18,000	0.092	fixed
	sigma	0.002	0.001	0.002	12,444		residual
**Habitat specificity - fluctuations CI**	(Intercept)	0.138	0.101	0.176	18,800	0.0001	fixed
	Habitat specificity	0.003	-0.001	0.006	18,000	0.137	fixed
	sigma	0.011	0.009	0.014	15,996		residual
**Habitat specificity - fluctuations CI weighted**	(Intercept)	0.146	0.098	0.192	18,046	0.0001	fixed
	Habitat specificity	0.003	-0.001	0.007	19,218	0.205	fixed
	sigma	0.010	0.008	0.013	15,033		residual
**Habitat specificity - fluctuations SE**	(Intercept)	0.155	0.114	0.198	18,000	0.0001	fixed
	Habitat specificity	0.001	-0.003	0.004	18,000	0.772	fixed
	sigma	0.009	0.007	0.010	18,000		residual
**Habitat specificity - fluctuations SD**	(Intercept)	0.400	0.360	0.438	17,592	0.0001	fixed
	Habitat specificity	0.0004	-0.003	0.004	18,000	0.844	fixed
	sigma	0.017	0.014	0.020	15,211		residual
**IUCN Red List Categories - *μ***	Least concern	0.013	0.003	0.023	17,332	0.011	fixed
	Near threatened	0.020	-0.014	0.053	18,436	0.259	fixed
	Vulnerable	0.014	-0.028	0.059	18,000	0.516	fixed
	Endangered	-0.038	-0.186	0.114	18,000	0.621	fixed
	Critically endangered	0.070	-0.024	0.166	17,353	0.148	fixed
	sigma	0.004	0.003	0.004	14,117		residual
**IUCN Red List Categories - weighted**	Least concern	0.017	0.006	0.029	17,546	0.003	fixed
	Near threatened	0.025	-0.017	0.064	16,702	0.222	fixed
	Vulnerable	0.001	-0.046	0.048	18,000	0.964	fixed
	Endangered	-0.038	-0.181	0.102	18,000	0.598	fixed

	Critically endangered	0.044	-0.200	0.283	16,964	0.713	fixed
	sigma	0.003	0.002	0.004	10,497		residual
**IUCN Red List Categories - slope**	Least concern	0.026	0.007	0.046	18,000	0.009	fixed
	Near threatened	0.016	-0.053	0.079	18,000	0.635	fixed
	Vulnerable	0.017	-0.069	0.102	18,000	0.689	fixed
	Endangered	-0.011	-0.289	0.275	18,091	0.934	fixed
	Critically endangered	0.053	-0.129	0.235	18,475	0.566	fixed
	sigma	0.011	0.009	0.013	15,903		residual
**IUCN Red List Categories - fluctuations *σ***	Least concern	0.073	0.056	0.090	19,089	0.0001	fixed
	Near threatened	0.052	-0.012	0.113	18,000	0.099	fixed
	Vulnerable	0.061	-0.018	0.141	18,461	0.139	fixed
	Endangered	0.173	-0.072	0.417	18,000	0.165	fixed
	Critically endangered	0.003	-0.159	0.166	18,000	0.976	fixed
	sigma	0.005	0.004	0.006	17,270		residual
**IUCN Red List Categories - fluctuations CI**	Least concern	0.165	0.144	0.187	18,000	0.0001	fixed
	Near threatened	0.134	0.057	0.211	18,000	0.001	fixed
	Vulnerable	0.140	0.041	0.240	18,000	0.005	fixed
	Endangered	0.237	-0.089	0.541	18,433	0.140	fixed
	Critically endangered	0.105	-0.103	0.314	17,573	0.321	fixed
	sigma	0.011	0.009	0.013	16,778		residual
**IUCN Red List Categories - fluctuations CI weighted**	Least concern	0.175	0.148	0.201	18,000	0.0001	fixed
	Near threatened	0.166	0.071	0.263	18,000	0.001	fixed
	Vulnerable	0.148	0.034	0.264	18,000	0.013	fixed
	Endangered	0.236	-0.100	0.568	18,000	0.176	fixed
	Critically endangered	0.070	-0.256	0.420	18,493	0.690	fixed
	sigma	0.010	0.008	0.012	15,655		residual
**IUCN Red List Categories - fluctuations SE**	Least concern	0.165	0.141	0.190	18,376	0.0001	fixed
	Near threatened	0.134	0.048	0.227	14,776	0.004	fixed
	Vulnerable	0.113	-0.002	0.228	17,971	0.051	fixed
	Endangered	0.071	-0.281	0.409	18,000	0.692	fixed
	Critically endangered	0.079	-0.156	0.315	18,000	0.516	fixed
	sigma	0.009	0.007	0.010	18,000		residual
**IUCN Red List Categories - fluctuations SD**	Least concern	0.408	0.386	0.429	18,770	0.0001	fixed

	Near threatened	0.358	0.287	0.432	18,000	0.0001	fixed
	Vulnerable	0.319	0.223	0.418	18,000	0.0001	fixed
	Endangered	0.243	-0.085	0.586	18,000	0.157	fixed
	Critically endangered	0.331	0.129	0.536	18,000	0.002	fixed
	sigma	0.016	0.015	0.016	18,000		residual

Supplementary Table 4. Phylogeny model outputs. To account for phylogenetic uncertainty, we ran the phylogenetic models for amphibian, bird and reptile species using 10 different trees for each class, and here we present the mean, min and max values from the different model runs. Sigma is the overall model residual variance. Net population change is estimated using μ values derived from state-space models of population abundance versus time and slopes of linear models of population abundance versus time. The fluctuation models were based on the process noise (σ^2) values from state-space models.

Model name	Variable	Mean pMCMC	Max pMCMC	Min pMCMC	Mean effective sample size	Mean post. mean	Max post. mean	Min post. mean	Mean lower 95% CI	Max lower 95% CI	Min lower 95% CI	Mean upper 95% CI	Max upper 95% CI	Min upper 95% CI
**Amphibian population trends**	(Intercept)	0.587	0.621	0.558	10047	-0.009	-0.008	-0.009	-0.051	-0.046	-0.054	0.031	0.035	0.027
	Phylogeny				7037	0.001	0.001	0.001	0	0	0	0.004	0.005	0.004
	Sigma				9856	0.006	0.006	0.006	0.004	0.004	0.004	0.007	0.007	0.007
	Species				7912	0.001	0.001	0	0	0	0	0.002	0.002	0.002
**Amphibian population fluctuations**	(Intercept)	0	0	0	9947	0.155	0.156	0.155	0.104	0.105	0.102	0.208	0.211	0.206
	Phylogeny				9778	0.001	0.001	0.001	0	0	0	0.005	0.006	0.005
	Sigma				10172	0.048	0.048	0.048	0.037	0.038	0.037	0.059	0.06	0.059
	Species				9817	0.001	0.001	0.001	0	0	0	0.003	0.003	0.003
**Bird population trends**	(Intercept)	0.449	0.63	0.305	10233	0.005	0.005	0.004	-0.009	-0.005	-0.014	0.018	0.021	0.016
	Phylogeny				3770	0	0	0	0	0	0	0	0.001	0
	Sigma				9992	0.002	0.002	0.002	0.002	0.002	0.002	0.003	0.003	0.003
	Species				6682	0	0	0	0	0	0	0	0	0
**Bird population fluctuations**	(Intercept)	0.001	0.006	0	10183	0.02	0.02	0.019	0.01	0.013	0.007	0.029	0.032	0.027
	Phylogeny				3995	0	0	0	0	0	0	0	0	0

	Sigma				9221	0.002	0.002	0.002	0.002	0.002	0.002	0.002	0.002	0.002
	Species				5322	0	0	0	0	0	0	0	0	0
**Reptile population trends**	(Intercept)	0.832	0.856	0.812	10030	0.004	0.005	0.003	-0.038	-0.033	-0.047	0.048	0.056	0.043
	Phylogeny				3971	0.002	0.002	0.001	0	0	0	0.007	0.01	0.005
	Sigma				3562	0.004	0.004	0.004	0.001	0.001	0.001	0.007	0.007	0.007
	Species				3419	0.004	0.004	0.004	0	0	0	0.007	0.007	0.007
**Reptile population fluctuations**	(Intercept)	0.003	0.004	0.001	9839	0.151	0.152	0.151	0.074	0.078	0.07	0.23	0.236	0.225
	Phylogeny				2958	0.004	0.005	0.004	0	0	0	0.017	0.019	0.016
	Sigma				3888	0.007	0.007	0.007	0.003	0.003	0.002	0.014	0.015	0.014
	Species				6057	0.043	0.043	0.043	0.025	0.026	0.024	0.062	0.064	0.061

Supplementary Table 5. List of species included in the UK scale analysis of population change across rarity metrics.

Species name	Number of populations
Acrocephalus schoenobaenus	1
Acrocephalus scirpaceus	1
Agonus cataphractus	1
Alca torda	4
Anarhichas lupus	1
Anas acuta	1
Anas crecca	1
Anas platyrhynchos	2
Anser albifrons	4
Anser fabalis	1
Anthus pratensis	2
Ardea cinerea	1
Arenaria interpres	1
Argentina silus	1
Argentina sphyraena	1
Arnoglossus laterna	1
Asio flammeus	1
Aythya ferina	4
Aythya fuligula	2
Botaurus stellaris	14
Branta bernicla	54
Branta canadensis	1
Branta leucopsis	3
Brosme brosme	1
Bucephala clangula	1
Bufo bufo	1
Burhinus oedicnemus	1
Buteo buteo	1
Calidris alba	1
Calidris alpina	1
Calidris canutus	2
Calidris maritima	2
Callionymus maculatus	1
Capreolus capreolus	3

Carduelis cannabina	2
Cepphus grylle	2
Cervus elaphus	2
Cetorhinus maximus	1
Cettia cetti	1
Charadrius hiaticula	1
Chelidonichthys lucerna	2
Circus aeruginosus	1
Circus cyaneus	1
Clupea harengus	2
Columba oenas	1
Coronella austriaca	1
Corvus corax	1
Corvus corone	1
Corvus monedula	1
Crex crex	1
Cyclopterus lumpus	1
Cygnus columbianus	3
Cygnus cygnus	1
Cygnus olor	1
Delphinus delphis	2
Echiichthys vipera	1
Egretta garzetta	1
Emberiza cirlus	1
Emberiza citrinella	1
Emberiza schoeniclus	3
Eptesicus serotinus	1
Esox lucius	2
Falco peregrinus	1
Falco tinnunculus	1
Fulica atra	1
Fulmarus glacialis	7
Gadus morhua	7
Glyptocephalus cynoglossus	1
Haematopus ostralegus	2
Haliaeetus albicilla	1
Halichoerus grypus	52
Hippoglossus hippoglossus	1

Lagopus lagopus	3
Larus argentatus	1
Larus canus	1
Larus fuscus	1
Larus melanocephalus	1
Lepidorhombus whiffiagonis	2
Lepus timidus	1
Limosa lapponica	2
Limosa limosa	2
Lissotriton vulgaris	5
Lophius budegassa	1
Lophius piscatorius	2
Lullula arborea	1
Melanogrammus aeglefinus	9
Meles meles	1
Mergus serrator	1
Merlangius merlangus	8
Merluccius merluccius	2
Micromesistius poutassou	1
Milvus milvus	3
Molva molva	1
Morus bassanus	4
Muscicapa striata	1
Myotis nattereri	1
Natrix natrix	1
Netta rufina	1
Numenius arquata	2
Nyctalus noctula	1
Oenanthe oenanthe	1
Orcinus orca	1
Oriolus oriolus	1
Oryctolagus cuniculus	6
Oxyura jamaicensis	2
Pandion haliaetus	1
Parus major	2
Passer domesticus	1
Passer montanus	1
Perca fluviatilis	3

Perdix perdix	1
Phalacrocorax aristotelis	8
Phoca vitulina	28
Phrynorhombus norvegicus	1
Pipistrellus pipistrellus	1
Pipistrellus pygmaeus	1
Platichthys flesus	1
Plecotus auritus	1
Plectrophenax nivalis	2
Pleuronectes platessa	6
Pluvialis apricaria	2
Pluvialis squatarola	1
Podiceps cristatus	1
Pollachius pollachius	1
Pollachius virens	3
Prunella modularis	1
Puffinus mauretanicus	2
Pyrrhula pyrrhula	1
Raja brachyura	1
Raja clavata	1
Raja microocellata	1
Raja montagui	1
Rhinolophus ferrumequinum	2
Rhinolophus hipposideros	4
Rissa tridactyla	9
Salmo salar	1
Salmo trutta	1
Scomber scombrus	2
Scyliorhinus canicula	1
Sitta europaea	1
Sprattus sprattus	1
Stenella coeruleoalba	1
Stercorarius parasiticus	3
Sterna dougallii	5
Sterna hirundo	1
Sterna paradisaea	1
Sternula albifrons	1
Streptopelia turtur	1

Strix aluco	1
Sturnus vulgaris	1
Sylvia communis	1
Syngnathus rostellatus	1
Tachybaptus ruficollis	1
Tadorna tadorna	1
Thalasseus sandvicensis	2
Trachurus trachurus	2
Tringa nebularia	1
Tringa totanus	1
Trisopterus esmarkii	2
Trisopterus luscus	1
Trisopterus minutus	1
Triturus cristatus	5
Turdus philomelos	1
Tursiops truncatus	4
Uria aalge	10
Vanellus vanellus	3
Xiphias gladius	1

Supplementary Table 6. Profiling method for estimating habitat specificity for 144 species with populations in the UK in the LPD. We extracted the habitats in which each species occurs from their IUCN Red List profiles (http://www.iucnredlist.org/) and we followed this key for consistency.

Habitat	Considered to be the same as:	Considered to be different to:
**Rural park**	Suburban park, urban park, rural garden, suburban garden, urban garden	
**Lake**	Big lake, small lake, pond, pool, dam, oxbow lake, reservoir	
**Bog**	Swamp, bogland	Lagoon
**Coastal cliff**	Island cliff	
**Shingle beach**	Pebble beach, rock beach	Sandy beach
**Stream**	River	Weir
**Fruit Tree Plantation**	Fruit Garden, orchard	
**Thicket**	Copse, grove, small stand	
**Forest**		Woodland
**Glade**	Forest Clearing	
**Broadleaf**	Deciduous	
**Urban**	Suburban	
**River margin**	Various types of river margins	
**Tidal Creek**		Estuary
**Harbour**	Dock, jetty, pier	
**Bush lands**	Shrublands	
**Irrigation channel**	Ditch	
**Heath**	Moorland	
**Sandy beach**	Spit, dune	Shingle beach, pebble beach, rock beach
**Crag**	Rocky outcrop, cliff, rocky slope	
**Marsh**	Wet meadow	
**Islet**	Island	

Supplementary Table 7. References for eighty time series (or 1% of analysed time series) which had very little variance (error < 0.001). See Supplementary Figure 6e for a visualisation of the data from a subsample of those time series.

Time series id	Data source citation
4178	Environment Canada (2015). North American Breeding Bird Survey - Canadian Trends Website. Data-version 2014. from http://www.ec.gc.ca/ron-bbs/P001/A001/?lang=e.
13773	Environment Canada (2015). North American Breeding Bird Survey - Canadian Trends Website. Data-version 2014. from http://www.ec.gc.ca/ron-bbs/P001/A001/?lang=e.
3697	Environment Canada (2015). North American Breeding Bird Survey - Canadian Trends Website. Data-version 2014. from http://www.ec.gc.ca/ron-bbs/P001/A001/?lang=e.
18118	Environment Canada (2015). North American Breeding Bird Survey - Canadian Trends Website. Data-version 2014. from http://www.ec.gc.ca/ron-bbs/P001/A001/?lang=e.
13797	Environment Canada (2015). North American Breeding Bird Survey - Canadian Trends Website. Data-version 2014. from http://www.ec.gc.ca/ron-bbs/P001/A001/?lang=e.
4218	Environment Canada (2015). North American Breeding Bird Survey - Canadian Trends Website. Data-version 2014. from http://www.ec.gc.ca/ron-bbs/P001/A001/?lang=e.
4220	Environment Canada (2015). North American Breeding Bird Survey - Canadian Trends Website. Data-version 2014. from http://www.ec.gc.ca/ron-bbs/P001/A001/?lang=e.
3725	Environment Canada (2015). North American Breeding Bird Survey - Canadian Trends Website. Data-version 2014. from http://www.ec.gc.ca/ron-bbs/P001/A001/?lang=e.
2916	Sauer, J. R., J. E. Hines, et al. (2012). The North American Breeding Bird Survey, Results and Analysis 1966 - 2011, USGS Patuxent Wildlife Research Center, Laurel, MD.
13846	Environment Canada (2015). North American Breeding Bird Survey - Canadian Trends Website. Data-version 2014. from http://www.ec.gc.ca/ron-bbs/P001/A001/?lang=e.
3771	Environment Canada (2015). North American Breeding Bird Survey - Canadian Trends Website. Data-version 2014. from http://www.ec.gc.ca/ron-bbs/P001/A001/?lang=e.
2987	Sauer, J. R., J. E. Hines, et al. (2012). The North American Breeding Bird Survey, Results and Analysis 1966 - 2011, USGS Patuxent Wildlife Research Center, Laurel, MD.
4236	Environment Canada (2015). North American Breeding Bird Survey - Canadian Trends Website. Data-version 2014. from http://www.ec.gc.ca/ron-bbs/P001/A001/?lang=e.
4237	Environment Canada (2015). North American Breeding Bird Survey - Canadian Trends Website. Data-version 2014. from http://www.ec.gc.ca/ron-bbs/P001/A001/?lang=e.
3803	Environment Canada (2015). North American Breeding Bird Survey - Canadian Trends Website. Data-version 2014. from http://www.ec.gc.ca/ron-bbs/P001/A001/?lang=e.
3808	Environment Canada (2015). North American Breeding Bird Survey - Canadian Trends Website. Data-version 2014. from http://www.ec.gc.ca/ron-bbs/P001/A001/?lang=e.
3816	Environment Canada (2015). North American Breeding Bird Survey - Canadian Trends Website. Data-version 2014. from http://www.ec.gc.ca/ron-bbs/P001/A001/?lang=e.
3813	Environment Canada (2015). North American Breeding Bird Survey - Canadian Trends Website. Data-version 2014. from http://www.ec.gc.ca/ron-bbs/P001/A001/?lang=e.

3821	Environment Canada (2015). North American Breeding Bird Survey - Canadian Trends Website. Data-version 2014. from http://www.ec.gc.ca/ron-bbs/P001/A001/?lang=e.
13944	Environment Canada (2015). North American Breeding Bird Survey - Canadian Trends Website. Data-version 2014. from http://www.ec.gc.ca/ron-bbs/P001/A001/?lang=e.
11350	KeiÅÁs, O. (2005). Impact of changes in agricultural land use on the Corncrake Crex crex population in Latvia. Acta Universitatis Latviensis 691: 93-109.
13976	Environment Canada (2015). North American Breeding Bird Survey - Canadian Trends Website. Data-version 2014. from http://www.ec.gc.ca/ron-bbs/P001/A001/?lang=e.
3997	Environment Canada (2015). North American Breeding Bird Survey - Canadian Trends Website. Data-version 2014. from http://www.ec.gc.ca/ron-bbs/P001/A001/?lang=e.
3995	Environment Canada (2015). North American Breeding Bird Survey - Canadian Trends Website. Data-version 2014. from http://www.ec.gc.ca/ron-bbs/P001/A001/?lang=e.
3998	Environment Canada (2014). North American Breeding Bird Survey - Canadian Trends Website. Data-version 2012. from http://www.ec.gc.ca/ron-bbs/P001/A001/?lang=e.
13694	Environment Canada (2015). North American Breeding Bird Survey - Canadian Trends Website. Data-version 2014. from http://www.ec.gc.ca/ron-bbs/P001/A001/?lang=e.
13520	Environment Canada (2015). North American Breeding Bird Survey - Canadian Trends Website. Data-version 2014. from http://www.ec.gc.ca/ron-bbs/P001/A001/?lang=e.
3018	Sauer, J. R., J. E. Hines, et al. (2012). The North American Breeding Bird Survey, Results and Analysis 1966 - 2011, USGS Patuxent Wildlife Research Center, Laurel, MD.
13687	Environment Canada (2015). North American Breeding Bird Survey - Canadian Trends Website. Data-version 2014. from http://www.ec.gc.ca/ron-bbs/P001/A001/?lang=e.
14076	Environment Canada (2015). North American Breeding Bird Survey - Canadian Trends Website. Data-version 2014. from http://www.ec.gc.ca/ron-bbs/P001/A001/?lang=e.
14074	Environment Canada (2015). North American Breeding Bird Survey - Canadian Trends Website. Data-version 2014. from http://www.ec.gc.ca/ron-bbs/P001/A001/?lang=e.
3326	Environment Canada (2015). North American Breeding Bird Survey - Canadian Trends Website. Data-version 2014. from http://www.ec.gc.ca/ron-bbs/P001/A001/?lang=e.
14095	Environment Canada (2015). North American Breeding Bird Survey - Canadian Trends Website. Data-version 2014. from http://www.ec.gc.ca/ron-bbs/P001/A001/?lang=e.
14093	Environment Canada (2015). North American Breeding Bird Survey - Canadian Trends Website. Data-version 2014. from http://www.ec.gc.ca/ron-bbs/P001/A001/?lang=e.
13584	Environment Canada (2015). North American Breeding Bird Survey - Canadian Trends Website. Data-version 2014. from http://www.ec.gc.ca/ron-bbs/P001/A001/?lang=e.
3883	Environment Canada (2015). North American Breeding Bird Survey - Canadian Trends Website. Data-version 2014. from http://www.ec.gc.ca/ron-bbs/P001/A001/?lang=e.
10449	Tofft, J. (2007). Tranens Grus grus bestandsudvikling i Danmark 1990-2006. Dansk Ornitologisk Forenings Tidsskrift 101(4): 67-72.
2579	Fylkesmannen i Vestfold (2004). Hekketakseringer, sjÌüfugl i Vestfold, MiljÌüvernavdelingen.
2632	Fylkesmannen i Vestfold (2004). Hekketakseringer, sjÌüfugl i Vestfold, MiljÌüvernavdelingen.
3360	Environment Canada (2015). North American Breeding Bird Survey - Canadian Trends Website. Data-version 2014. from http://www.ec.gc.ca/ron-bbs/P001/A001/?lang=e.

14286	Environment Canada (2015). North American Breeding Bird Survey - Canadian Trends Website. Data-version 2014. from http://www.ec.gc.ca/ron-bbs/P001/A001/?lang=e.
4245	Environment Canada (2014). North American Breeding Bird Survey - Canadian Trends Website. Data-version 2012. from http://www.ec.gc.ca/ron-bbs/P001/A001/?lang=e.
4247	Environment Canada (2015). North American Breeding Bird Survey - Canadian Trends Website. Data-version 2014. from http://www.ec.gc.ca/ron-bbs/P001/A001/?lang=e.
18111	Environment Canada (2015). North American Breeding Bird Survey - Canadian Trends Website. Data-version 2014. from http://www.ec.gc.ca/ron-bbs/P001/A001/?lang=e.
4229	Environment Canada (2015). North American Breeding Bird Survey - Canadian Trends Website. Data-version 2014. from http://www.ec.gc.ca/ron-bbs/P001/A001/?lang=e.
1037	Environment Canada (2015). North American Breeding Bird Survey - Canadian Trends Website. Data-version 2014. from http://www.ec.gc.ca/ron-bbs/P001/A001/?lang=e.
14728	Environment Canada (2015). North American Breeding Bird Survey - Canadian Trends Website. Data-version 2014. from http://www.ec.gc.ca/ron-bbs/P001/A001/?lang=e.
14208	Environment Canada (2015). North American Breeding Bird Survey - Canadian Trends Website. Data-version 2014. from http://www.ec.gc.ca/ron-bbs/P001/A001/?lang=e.
3947	Environment Canada (2015). North American Breeding Bird Survey - Canadian Trends Website. Data-version 2014. from http://www.ec.gc.ca/ron-bbs/P001/A001/?lang=e.
4254	Environment Canada (2015). North American Breeding Bird Survey - Canadian Trends Website. Data-version 2014. from http://www.ec.gc.ca/ron-bbs/P001/A001/?lang=e.
4253	Environment Canada (2015). North American Breeding Bird Survey - Canadian Trends Website. Data-version 2014. from http://www.ec.gc.ca/ron-bbs/P001/A001/?lang=e.
14217	Environment Canada (2014). North American Breeding Bird Survey - Canadian Trends Website. Data-version 2012. from http://www.ec.gc.ca/ron-bbs/P001/A001/?lang=e.
3958	Environment Canada (2015). North American Breeding Bird Survey - Canadian Trends Website. Data-version 2014. from http://www.ec.gc.ca/ron-bbs/P001/A001/?lang=e.
3245	Sauer, J. R., J. E. Hines, et al. (2012). The North American Breeding Bird Survey, Results and Analysis 1966 - 2011, USGS Patuxent Wildlife Research Center, Laurel, MD.
14249	Environment Canada (2015). North American Breeding Bird Survey - Canadian Trends Website. Data-version 2014. from http://www.ec.gc.ca/ron-bbs/P001/A001/?lang=e.
6515	Herrero, M. A. N. (2006). Results of a 10-years (1994-2003) monitoring Programme of Shore Birds Populations in the Protected landscape of Rambla Salada and Ajauque (Inner Saltworks) in Murcia, Spain. A Contribution for 2010 Biodiversity Index.
4261	Environment Canada (2014). North American Breeding Bird Survey - Canadian Trends Website. Data-version 2012. from http://www.ec.gc.ca/ron-bbs/P001/A001/?lang=e.
14327	Environment Canada (2015). North American Breeding Bird Survey - Canadian Trends Website. Data-version 2014. from http://www.ec.gc.ca/ron-bbs/P001/A001/?lang=e.
3674	Environment Canada (2015). North American Breeding Bird Survey - Canadian Trends Website. Data-version 2014. from http://www.ec.gc.ca/ron-bbs/P001/A001/?lang=e.
13636	Environment Canada (2015). North American Breeding Bird Survey - Canadian Trends Website. Data-version 2014. from http://www.ec.gc.ca/ron-bbs/P001/A001/?lang=e.

4051	Environment Canada (2015). North American Breeding Bird Survey - Canadian Trends Website. Data-version 2014. from http://www.ec.gc.ca/ron-bbs/P001/A001/?lang=e.
4069	Environment Canada (2015). North American Breeding Bird Survey - Canadian Trends Website. Data-version 2014. from http://www.ec.gc.ca/ron-bbs/P001/A001/?lang=e.
4086	Environment Canada (2015). North American Breeding Bird Survey - Canadian Trends Website. Data-version 2014. from http://www.ec.gc.ca/ron-bbs/P001/A001/?lang=e.
4088	Environment Canada (2015). North American Breeding Bird Survey - Canadian Trends Website. Data-version 2014. from http://www.ec.gc.ca/ron-bbs/P001/A001/?lang=e.
4083	Environment Canada (2015). North American Breeding Bird Survey - Canadian Trends Website. Data-version 2014. from http://www.ec.gc.ca/ron-bbs/P001/A001/?lang=e.
3119	Sauer, J. R., J. E. Hines, et al. (2012). The North American Breeding Bird Survey, Results and Analysis 1966 - 2011, USGS Patuxent Wildlife Research Center, Laurel, MD.
4085	Environment Canada (2015). North American Breeding Bird Survey - Canadian Trends Website. Data-version 2014. from http://www.ec.gc.ca/ron-bbs/P001/A001/?lang=e.
4087	Environment Canada (2015). North American Breeding Bird Survey - Canadian Trends Website. Data-version 2014. from http://www.ec.gc.ca/ron-bbs/P001/A001/?lang=e.
4093	Environment Canada (2015). North American Breeding Bird Survey - Canadian Trends Website. Data-version 2014. from http://www.ec.gc.ca/ron-bbs/P001/A001/?lang=e.
5700	Bailey, K. M. and S. A. Macklin (1994). Analysis of patterns in larval walleye pollpck Theragra chalcogramma survival and wind mixing events in Shelikof Strait Gulf of Alaska. Marine Ecology Progress Series 113: 1-12.
4099	Environment Canada (2015). North American Breeding Bird Survey - Canadian Trends Website. Data-version 2014. from http://www.ec.gc.ca/ron-bbs/P001/A001/?lang=e.
8476	Lee, P.-F., I. C. Chen, et al. (2005). Spatial and temporal distribution patterns of bigeye tuna (Thunnus obesus) in the Indian Ocean. Zoological Studies 44(2): 260-270.
14807	Environment Canada (2015). North American Breeding Bird Survey - Canadian Trends Website. Data-version 2014. from http://www.ec.gc.ca/ron-bbs/P001/A001/?lang=e.
14801	Environment Canada (2015). North American Breeding Bird Survey - Canadian Trends Website. Data-version 2014. from http://www.ec.gc.ca/ron-bbs/P001/A001/?lang=e.
14804	Environment Canada (2015). North American Breeding Bird Survey - Canadian Trends Website. Data-version 2014. from http://www.ec.gc.ca/ron-bbs/P001/A001/?lang=e.
4132	Environment Canada (2015). North American Breeding Bird Survey - Canadian Trends Website. Data-version 2014. from http://www.ec.gc.ca/ron-bbs/P001/A001/?lang=e.
4143	Environment Canada (2015). North American Breeding Bird Survey - Canadian Trends Website. Data-version 2014. from http://www.ec.gc.ca/ron-bbs/P001/A001/?lang=e.
4139	Environment Canada (2015). North American Breeding Bird Survey - Canadian Trends Website. Data-version 2014. from http://www.ec.gc.ca/ron-bbs/P001/A001/?lang=e.
4151	Environment Canada (2015). North American Breeding Bird Survey - Canadian Trends Website. Data-version 2014. from http://www.ec.gc.ca/ron-bbs/P001/A001/?lang=e.
18190	Environment Canada (2015). North American Breeding Bird Survey - Canadian Trends Website. Data-version 2014. from http://www.ec.gc.ca/ron-bbs/P001/A001/?lang=e.

259

Supplementary Table 8. References for time series which appear to show logistic growth with little variance (see Supplementary Figure 6e for visualisation of data).

Time series id	Data source citation
468	NERC Centre for Population Biology (1999). The Global Populations Dynamics Database. http://cpbnts1.bio.ic.ac.uk/gpdd/, Imperial College,.l Batten, L. A. and J. H. Marchant (1977). Bird Population Changes for Years 1974-75. Bird Study 24(1): 55-61.
17803	Government of Antigua and Barbuda (2014). Antigua and Barbuda Fifth National Report to the Convention on Biodiversity, Environment Division: 1-66.
10193	Giling, D., R. D. Reina, et al. (2008). Anthropogenic influence on an urban colony of the little penguin Eudyptula minor. Marine and Freshwater Research 59(7): 647-651.

References

1. Humbert, J.-Y., Scott Mills, L., Horne, J. S. & Dennis, B. A better way to estimate population trends. *Oikos* **118**, 1940–1946 (2009).
2. van de Pol, M. & Wright, J. A simple method for distinguishing within- versus between-subject effects using mixed models. *Animal Behaviour* **77**, 753–758 (2009).
3. Hadfield, J. D. MCMC methods for multi-response generalized linear mixed models: the MCMCglmm R package. *Journal of Statistical Software* **33**, 1–22 (2010).
4. Fournier, A. M. V., White, E. R. & Heard, S. B. Site-selection bias and apparent population declines in long-term studies. *Conservation Biology* **33**, 1370–1379 (2019).
