# Supplementary material for: Rare and common vertebrates span a wide spectrum of population trends
Source: Nat Commun. 2020 Sep 2;11:4394. doi: 10.1038/s41467-020-17779-0 (PMC7468135; doi:10.1038/s41467-020-17779-0)
Supplement: Supplementary file 3 — Reporting Summary [file 41467_2020_17779_MOESM3_ESM.pdf]

nature research

Corresponding author(s): Gergana Daskalova

Reporting Summary

Nature Research wishes to improve the reproducibility of the work that we publish. This form provides structure for consistency and transparency in reporting. For further information on Nature Research policies, see Authors & Referees and the Editorial Policy Checklist.

Statistical parameters

When statistical analyses are reported, confirm that the following items are present in the relevant location (e.g. figure legend, table legend, main text, or Methods section).

n/a	Confirmed	
☐	☒	The exact sample size (*n*) for each experimental group/condition, given as a discrete number and unit of measurement
☐	☒	An indication of whether measurements were taken from distinct samples or whether the same sample was measured repeatedly
☐	☒	The statistical test(s) used AND whether they are one- or two-sided<br>*Only common tests should be described solely by name; describe more complex techniques in the Methods section.*
☐	☒	A description of all covariates tested
☐	☒	A description of any assumptions or corrections, such as tests of normality and adjustment for multiple comparisons
☐	☒	A full description of the statistics including central tendency (e.g. means) or other basic estimates (e.g. regression coefficient) AND variation (e.g. standard deviation) or associated estimates of uncertainty (e.g. confidence intervals)
☐	☒	For null hypothesis testing, the test statistic (e.g. *F*, *t*, *r*) with confidence intervals, effect sizes, degrees of freedom and *P* value noted<br>*Give P values as exact values whenever suitable.*
☐	☒	For Bayesian analysis, information on the choice of priors and Markov chain Monte Carlo settings
☐	☒	For hierarchical and complex designs, identification of the appropriate level for tests and full reporting of outcomes
☐	☒	Estimates of effect sizes (e.g. Cohen's *d*, Pearson's *r*), indicating how they were calculated
☐	☒	Clearly defined error bars<br>*State explicitly what error bars represent (e.g. SD, SE, CI)*

Our web collection on statistics for biologists may be useful.

Software and code

Policy information about availability of computer code

Data collection	All R code used to download and integrate the different open-source databases we used in our analyses is deposited in a GitHub repository. We are happy to grant reviewers and editors access to the repository, and we will make all R code available upon publication. We have archived a library of all of the R packages we used in the specific versions upon which the data collection and database integration are based, and this archive is also included in our GitHub repository.
Data analysis	All R code used for our analyses is deposited in a GitHub repository (https://github.com/gndaskalova/PopChangeRarity, DOI 10.5281/zenodo.3817207).<br><br>All statistical analyses were conducted using R version 3.5.1.

For manuscripts utilizing custom algorithms or software that are central to the research but not yet described in published literature, software must be made available to editors/reviewers upon request. We strongly encourage code deposition in a community repository (e.g. GitHub). See the Nature Research guidelines for submitting code & software for further information.

Data

Policy information about availability of data

All manuscripts must include a data availability statement. This statement should provide the following information, where applicable:

- Accession codes, unique identifiers, or web links for publicly available datasets
- A list of figures that have associated raw data
- A description of any restrictions on data availability

Raw data are available from the following websites: for population time series - http://www.livingplanetindex.org/data_portal, GBIF occurrences - https://www.gbif.org, bird geographic ranges - http://datazone.birdlife.org, mammal geographic ranges - http://esapubs.org/archive/ecol/E090/184/, species' habitat preferences, threat types and IUCN Red List Categories - https://www.iucnredlist.org, and phylogenies - https://vertlife.org and https://birdtree.org.

Field-specific reporting

Please select the best fit for your research. If you are not sure, read the appropriate sections before making your selection.

☐ Life sciences ☐ Behavioural & social sciences ☒ Ecological, evolutionary & environmental sciences

For a reference copy of the document with all sections, see nature.com/authors/policies/ReportingSummary-flat.pdf

Ecological, evolutionary & environmental sciences study design

All studies must disclose on these points even when the disclosure is negative.

Study description	Our study aimed to determine if variation in vertebrate population change (trends and fluctuations) is explained by differences in species attributes, such as rarity metrics (geographic range, mean population size and habitat specificity) and conservation status.<br><br>A list of all analyses undertaken is available in Supplementary Figure 1 and the structure of our models (including all model terms and their estimates) is presented in Supplementary Tables 2 and 5.<br><br>We did not include random effects in the Bayesian models (e.g., a species or region random intercept), because only 28% of the studied species had more than four monitored populations and spatial replication was low (Supplementary Table 1).
Research sample	We extracted the abundance data for 9284 populations from 2084 vertebrate species from the Living Planet Database (see Table S3 for a list of all species included in our UK-scale analyses, a list of all species in the global analyses is available upon request). We downloaded occurrence data from the Global Biodiversity Information Facility and BirdLife, and information on species habitat preferences and conservation status from the IUCN Red List. We obtained phylogenies for amphibian species from Jetx et al. 2018, for bird species from Jetz et al. 2012, and for reptile species from Tonini et al. 2016.<br><br>LPI. Living Planet Index database, 2016. www.livingplanetindex.org/ Downloaded February 2016. (2016).<br>GBIF. Biodiversity occurrence data. http://www.gbif.org/ Downloaded Feb 2018. (2018).<br>BirdLife International. IUCN Red List for birds. Downloaded from http://www.birdlife.org in March 2018. (2018).<br>IUCN. Conservation status data. http://www.iucnredlist.org/ Downloaded Jan- April 2017. (2017).<br>Jetz, W. & Pyron, R. A. The interplay of past diversification and evolutionary isolation with present imperilment across the amphibian tree of life. Nat. Ecol. Evol. (2018). doi:10.1038/s41559-018-0515-5<br>Jetz, W., Thomas, G. H., Joy, J. B., Hartmann, K. & Mooers, A. O. The global diversity of birds in space and time. Nature 491, 444–448 (2012).<br>Tonini, J. F. R., Beard, K. H., Ferreira, R. B., Jetz, W. & Pyron, R. A. Fully- sampled phylogenies of squamates reveal evolutionary patterns in threat status. Biol. Conserv. 204, 23–31 (2016).
Sampling strategy	Sample size was chosen based on the amount of populations which had at least five time points of monitoring data. Since we were working with open-source database, our sample size was further determined by the amount of temporal population data that are publicly available as part of the Living Planet Database. Our sample size of 9284 populations from 2084 species represents the marine, terrestrial and freshwater realms and has a global extent. The species come from five vertebrate taxa (Actinopterygii, Amphibia, Aves, Mammalia, Reptilia). Sample size differed across our different analyses (see Table S1 for the number of populations and species included in each analysis) due to data availability (e.g., range size data were not available for all species). We did not use statistical methods to predetermine sample size. Instead, we worked with data from the most comprehensive open source databases.
Data collection	We used data that were collected by a range of scientists and organisations as all of the data we worked with are open access. The details for data collection for each specific dataset are available from their respective links (see Data section of the reporting summary). We integrated several open-source databases, which each included records collected by different people and using a variety of methods. Note that methods were standardised on the population level, i.e., monitoring methods differed among populations, but each population was survered using a consistent protocol. For details, please see the reference list outlined above in "Research sample".
Timing and spatial scale	Monitoring start and end differed among populations, but overall the Living Planet Database spans the period between 1970 and 2014. The spatial scale of our analyses is global, but there are regions of the world from where long-term population data are not

available. The duration and frequency of monitoring varied among populations (see Supplementary Figure 8). For a geographical representations of our sample size, see Figure 2a. The rest of the datasets we used were not temporal since they refer to static characteristics of a species (e.g., habitat specificity) and thus do not have associated durations and monitoring frequencies. We worked with open-access data collected by other people - for the original rationale behind the data collection, please see the original data references.

Data exclusions	In our analyses, we retained only populations that had at least five time points of monitoring data. Monitoring duration differed among populations (Supplementary Figures 1 and 2, see Supplementary Figures 5 and 9 for effects of monitoring duration on detected trends).<br><br>We filtered the GBIF data to remove invalid records and outliers using the CoordinateCleaner package. We followed pre-defined exclusion criteria - we excluded records with no decimal places in the decimal latitude or longitude values, with equal latitude or longitude, within a one degree radius of the GBIF headquarters in Copenhagen, within 0.0001 degrees of various biodiversity institutions and within 0.1 degrees of capital cities. For each species, we excluded the lower 0.02 and upper 0.98 quantile intervals of the latitude and longitude records to account for outlier points that are records from zoos or other non-wild populations.
Reproducibility	Throughout our analysis, we followed a reproducible coding framework. All analyses can be reproduced following our R code (note that the estimates from the Bayesian models will slightly differ every time the models are reran due to MCMC error).
Randomization	We followed taxonomic and ecological organisation, which was the focus of some of our research questions. Our sample populations come from five different taxa and occupy different biomes and habitats. We tested for taxa, biome and habitat effects on population change (see Figures 2-3).
Blinding	The study did not involve blinding, as no experiments or field data collection were undertaken and our aim was to include the most representative sample of temporal vertebrate population records possible.

Did the study involve field work? ☐ Yes ☒ No

Reporting for specific materials, systems and methods

Materials & experimental systems

n/a	Involved in the study
☒	☐ Unique biological materials
☒	☐ Antibodies
☒	☐ Eukaryotic cell lines
☒	☐ Palaeontology
☐	☒ Animals and other organisms
☒	☐ Human research participants

Methods

n/a	Involved in the study
☒	☐ ChIP-seq
☒	☐ Flow cytometry
☒	☐ MRI-based neuroimaging

Animals and other organisms

Policy information about studies involving animals; ARRIVE guidelines recommended for reporting animal research

Laboratory animals	The study did not involve laboratory animals.
Wild animals	The authors did not conduct fieldwork and did not directly work with individuals from the populations studied here. For details on the Living Planet Database, please see http://www.livingplanetindex.org/data_portal .
Field-collected samples	The study did not involve field-collected samples by the authors. For details on the Living Planet Database, please see http://www.livingplanetindex.org/data_portal .
